# Supplementary material for: An unusual type of polymorphism in a liquid crystal
Source: Nat Commun. 2018 Feb 19;9:714. doi: 10.1038/s41467-018-03160-9 (PMC5818537; doi:10.1038/s41467-018-03160-9)
Supplement: Supplementary file 1 — Supplementary Information [file 41467_2018_3160_MOESM1_ESM.pdf]

Supplementary Information

for

An unusual type of polymorphism in a liquid crystal

Li et al.

Supplementary Note 1. Materials

Commercial available chemicals were used as purchased without any further purification. All organic solvents used for synthesis were EMD Millipore grade purified by a PureSolv solvent purification system (Innovative Technology Inc.) unless otherwise stated. Dichloromethane (CH_2Cl_2) and benzene (C_6H_6) for syntheses were dried before use with 4-Å molecular sieves overnight. Tetrahydrofuran (THF) was dried over a sodium-benzophenone and distilled under a nitrogen atmosphere prior to use. All glassware used for the reactions was dried overnight at 140 °C in an oven. All reagents used were purchased from Sigma Aldrich except for 4-*n*-octyloxybiphenyl-4'-carboxylic acid (purchased from Synthon Chemicals GmbH & Co. KG) and for 1-ethyl-3-(3-dimethylaminopropyl)carbodiimide (EDCI) (purchased from TCI).

Supplementary Note 2. General experimental methods

^{1}H NMR (400 MHz) and ^{13}C NMR (100 MHz) spectra for synthesized materials were obtained using Bruker Avance 400 MHz spectrometer in $CDCl_3$. ^{1}H NMR spectra are reported in parts per million (*δ*) relative to residual solvent peaks (7.26 ppm for $CDCl_3$ and 0.08 ppm for tetramethyl-silane (TMS)) ^{13}C NMR spectra are reported in parts per million (*δ*) relative to residual solvent peaks (77.00 for $CDCl_3$).

Supplementary Note 3. Synthesis

The (8, 7+1*R*)-tris-BiPh diester was synthesized using the following synthetic route.

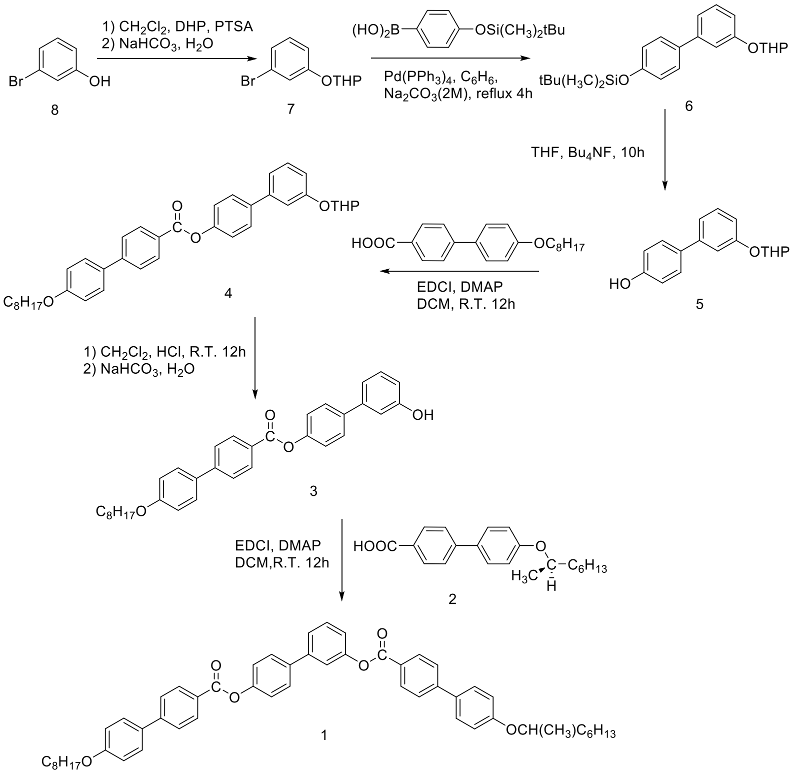

Supplementary Figure 1. Synthetic route pursued to obtain compound **1**, the (8, 7+1*R*)-tris-BiPh diester.

3.1 3-Tetrahydropyranyloxybromobenzene **7**

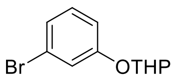

7

In a round-bottomed flask, 3-bromophenol (1.756 g, 10 mmol) was dissolved with 10 mL CH_2Cl_2 and cooled with an ice bath. 3,4-Dihydro-2H-pyrane (1.041 g, 12 mmol) was added dropwise over 10 min at 0 °C while stirring. After solution became clear, *p*-toluenesulfonic acid monohydrate (1.93 mg, 0.01 mmol) was added. The solution was stirred for 15 min at 20 °C and examined by TLC. Then it was quenched with addition of $NaHCO_3$ (0.118 g, 1.4 mmol) and 3 drops of water by stirring for 5 min. The solvent was removed by rotary evaporation under reduced pressure. The residue was purified by column chromatography with $CHCl_3$ and dried in a vacuum oven to yield a colorless liquid (2.2 g, 85%).

^{1}H NMR ($CDCl_3$, 400 MHz, δ/ppm): δ 7.27-7.29 (m, 1H, Ar-H), 7.13-7.18 (m, 2H, Ar-H), 7.01 (dt, 1H, J = 6.95Hz, 2.56 Hz, Ar-H), 5.43(t, 1H, J = 3.11 Hz, -OC$\underline{H}$(CH_2-)-O-, THP), 3.87-3.92(m, 1 H, -O-C$\underline{H_a}$$H_b$-$CH_2$-, THP), 3.67-3.62 (m, -O-C$H_a$$\underline{H_b}$-$CH_2$-, THP), 1.97-2.07 (m, 1H, THP), 1.86-1.90 (m, 2H, THP), 1.58-1.77 (m, 3H, THP).

3.2 4'-(*tert*-Butyldimethylsilyloxy)-3-tetrahydropyranyloxybiphenyl **6**

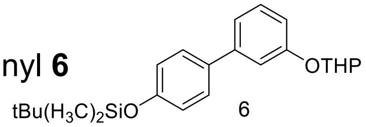

To a flask with benzene (6 mL) and aqueous $NaCO_3$ (2M, 6 mL) at room temperature under N_2 atmosphere, 3-tetrahydropyranyloxybromobenzene **7** (1.213 g, 4.72 mmol) was added followed by addition of 4-(*tert*-butyldimethylsilyloxy)phenylboronic acid (1.0 g, 3.97 mmol) and $Pd(PPh_3)_4$ (138.1 mg, 0.119 mmol). The mixture was heated to reflux with stirring for 4 hours with solution color from golden to red and examined by TLC. The solution was then extracted with CH_2Cl_2 and dried with Na_2SO_4. The solvent was removed by rotary evaporation under reduced pressure. The residue was purified by column chromatography with $CHCl_3$, recrystallized form *n*-hexane, and dried in a vacuum oven to yield a yellow oil (400 mg, 20%).

^{1}H NMR ($CDCl_3$, 400 MHz, δ/ppm): δ 7.40-7.60 (m, 2H, biphenyl), 7.27-7.36 (m, 1H, biphenyl), 7.23-7.26 (m, 1H, biphenyl), 7.04-7.22 (m, 1H, biphenyl), 6.97-7.01 (m, 1qH, biphenyl), 6.89 (dt, 1H, J = 8.42 Hz, 2.93 Hz, biphenyl), 5.39-5.49 (m, 1H, -OC$\underline{H}$(CH_2-)-O-, THP), 3.84-3.98 (m, 1H, -O-C$\underline{H_a}$$H_b$-$CH_2$-, THP), 3.59-3.65 (m, 1H, -O-C$H_a$$\underline{H_b}$-$CH_2$-, THP), 1.93-2.07 (m, 1H, THP), 1.83-1.92 (m, 2H, THP), 1.58-1.75 (m, 3H, THP), 1.01 (s, 9H, -Si-$C_3\underline{H_9}$-), 0.23 (s, 6H, -Si(C$\underline{H_3}$)$_2$-).

3.3 4'-Hydroxy-3-tetrahydropyranyloxybiphenyl **5** or 3'-(tetrahydro-2H-pyran-2-yloxy)biphenyl-4-ol

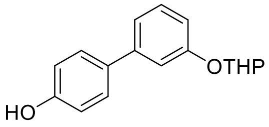

5

4'-(*tert*-Butyldimethylsilyloxy)-3-tetrahydropyranyloxybiphenyl **6** (400 mg, 0.78 mmol) was dissolved in THF (10 mL) and then tetrabutylammonium fluoride hydrate $Bu_4NF \cdot xH_2O$ (416.2 mg, 1.56 mmol) was then added. The solution was stirred at room temperature for 10 hours and examined by TLC. The solvent was removed by rotary evaporation under reduced pressure. The residue was dissolved in $CHCl_3$, washed with water, and dried with Na_2SO_4. The product was purified by column chromatography (petroleum ether / ethyl acetate = 10 / 4) and dried in a vacuum oven to yield light yellow solid (176 mg, 83%).

^{1}H NMR ($CDCl_3$, 400 MHz, *δ*/ppm): δ 7.47 (dt, *J* = 8.78, 2.93 Hz, 2 H, biphenyl), 7.32 (t, 1H, *J* = 7.32 Hz, biphenyl), 7.24 (t, 1H, *J* = 1.83 Hz, biphenyl), 7.17 (dq, 1H, *J* = 7.32, 0.73 Hz, biphenyl), 7.00 (dq, 1H, *J* = 8.05, 0.73 Hz, biphenyl), 6.88 (dt, 2H, *J* = 8.42, 2.93 Hz, biphenyl), 5.50 (t, 1H, *J* = 3.29 Hz, -OCH(CH_2-)-O-, THP), 3.93-3.99 (m, 1H, -O-CH_aH_b-CH_2-, THP), 3.61-3.66 (m, 1H, -O-CH_aH_b-CH_2-, THP), 1.87-1.91 (m, 2H, THP), 1.64-1.75 (m, 3H, THP).

3.4 4’-[4-(4-Octyloxy)biphenyloxy]-3-tetrahydropyranyloxybiphenyl **4**

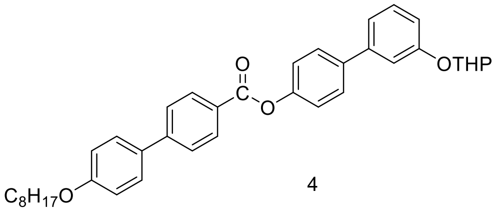

In a N_2-purged flask, 4’-hydroxy-3-tetrahydropyranyloxybiphenyl (140.6 mg, 0.52 mmol), 4-*n*-octyloxybiphenyl-4’-carboxylic acid (220.7 mg, 0.68 mmol), and 4-(dimethylamino)pyridine (DMAP) (139.8 mg, 1.14 mmol) was dissolved in a mixture of dichloromethane (5 mL) and THF (5 mL) and stirred for 5 minutes. To this mixture was added EDCI (299.1 mg, 1.56 mmol). The reaction was kept under stirring at room temperature under N_2 for 24 hours and examined by TLC. The resulting mixture was washed with saturated NaCl solution, extracted with $CHCl_3$, and then dried over anhydrous Na_2SO_4. The filtrate was collected by filtration and concentrated by rotary evaporation under reduced pressure. The residue was deposited onto silica and purified by column chromatography with $CHCl_3$ to yield a white solid (51 mg, 17%).

^{1}H NMR ($CDCl_3$, 400 MHz, *δ*/ppm): δ 8.27 (dt, 2H, *J* = 8.42, 1.80 Hz, biphenyl), 7.72 (dt, 2H, *J* = 8.78, 1.80 Hz, biphenyl), 7.66 (dt, 2H, *J* = 8.42, 2.93 Hz, biphenyl), 7.61 (dt, 2H, biphenyl *J* = 8.78, 2.20 Hz,), 7.37 (t, 1H, *J* = 7.68 Hz, biphenyl), 7.29-7.32 (m, 3H, biphenyl), 7.24 (dt, 1H, *J* = 7.7, 1.1 Hz, biphenyl), 7.06-7.09 (m, 1H, biphenyl), 7.02 (dt, 2 H, *J* = 8.78, 2.93 Hz, biphenyl), 5.51 (t, 1H, *J* = 3.11 Hz, -OCH(CH_2-)-O-, THP), 4.03 (t, 2H, *J* = 6.59 Hz, -OCH_2-C_7H_{15}), 3.94-4.00 (m, 1H, -O-CH_aH_b-CH_2-, THP), 3.62-3.67 (m, 1H, -O-CH_aH_b-CH_2-, THP), 1.80-1.93 (m, 3+2H, THP, -OC_2H_4-CH_2-C_5H_{11}), 1.62-1.75 (m, 3H, THP), 1.46-1.53 (m, 2H, -OCH_2-CH_2-C_6H_{13}), 1.28-1.41 (m, 8H, -OC_3H_6-C_4H_8-CH_3), 0.91 (t, 1H, *J* = 6.95 Hz, -CH_3). ^{13}C NMR ($CDCl_3$, 100 MHz, *δ*/ppm): 165.14, 159.58, 151.43, 150.73, 146.05, 142.07, 137.97, 131.93, 130.74, 129.84, 128.38, 128.30, 127.42, 127.38, 126.61, 124.60, 122.12, 120.50, 114.98, 59.16, 34.03, 31.81, 30.31, 29.69, 29.35, 29.25, 26.90, 26.05, 22.66, 21.87, 14.11, 13.90.

3.5 4’-[4-(4-Octyloxy)biphenyloxy]-3-hydroxybiphenyl **3**

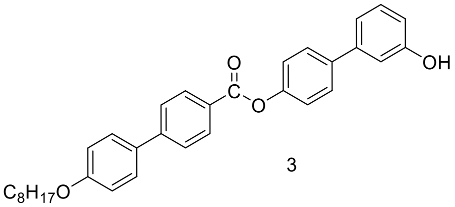

4’-[4-(4-Octyloxy)biphenyloxy]-3-tetrahydropyranyloxybiphenyl **4** (51.4 mg, 0.09 mmol) was dissolved in CH_2Cl_2 (6.5 mL) followed by addition of HCl (33%, 81 μL). The solution was stirred at room temperature for 12 hours. To this mixture, $NaHCO_3$ (0.25 g) and water (10 mL) were added. The solid was filtered off and washed with $CHCl_3$. The organic phase was dried over anhydrous Na_2SO_4. The filtrate was collected by filtration and thereafter concentrated by rotary evaporation under reduced pressure. The residue was purified by column chromatography ($CHCl_3$ / MeOH = 10 / 0.5) to yield a white solid (28 mg, 64%).

^{1}H NMR ($CDCl_3$, 400 MHz, *δ*/ppm): δ 8.26 (dt, 2H, *J* = 8.78 Hz, 1.46 Hz, biphenyl), 7.70 (dt, 2H, *J* = 8.78 HZ, 1.93 Hz, biphenyl), 7.59-7.64 (m, 3H, biphenyl), 7.45-7.52 (m, 2H, biphenyl), 7.30

(dt, 2H, J = 8.4 Hz, 2.2 Hz, biphenyl), 7.16-7.19 (m, 2H, biphenyl), 7.06-7.07 (m, 1H, biphenyl), 7.01 (dt, 2H, J = 7.78 Hz, 2.93 Hz, biphenyl), 4.02 (t, 2H, J = 6.6 Hz, $-OC\underline{H_2}C_7H_{15}$), 1.80-1.90 (m, 2H, $-C\underline{H_2}C_6H_{13}$), 1.45-1.52 (m, 2H, $-C\underline{H_2}C_5H_{11}$), 1.28-1.39 (m, 8H, $-C_4\underline{H_8}CH_3$), 0.90 (t, 3H, $-C\underline{H_3}$). ^{13}C NMR ($CDCl_3$, 100 MHz, δ/ppm): 159.61, 155.92, 155.44, 150.59, 146.08, 142.19, 130.74, 130.03, 129.71, 128.47, 128.38, 128.18, 126.61, 122.01, 120.03, 119.92, 115.70, 115.01, 114.28, 114.08, 68.19, 32.54, 29.69, 29.35, 29.25, 26.05, 21.87, 13.88

3.6 4'-[2-(*R*)-heptan-2-yloxy]biphenyl-4-carboxylic acid **2**

The synthesis of compound **2** was reported previously.[1]

3.7 4'-[4-(4'-Octyloxy)biphenyloxy]-3-[4-(4'-[2-(*R*)-heptan-2-yloxy]biphenyl)]-biphenyl **1**

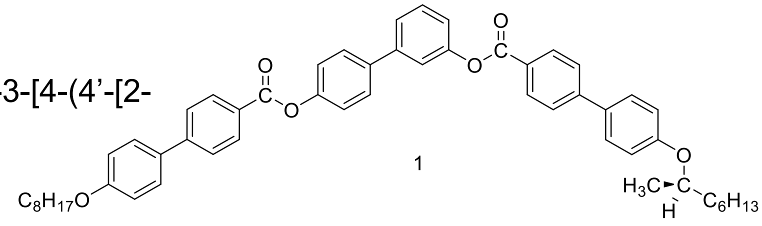

1

4'-[4-(4'-Octyloxy)biphenyloxy]-3-[4-(4'-[2-(*R*)-heptan-2-yloxy]biphenyl)]biphenyl **1** was prepared by the same synthetic method as 4'-[4-(4-octyloxy)biphenyloxy]-3-tetrahydropyranyloxybiphenyl **4**. Quantities: 4'-[4-(4-octyloxy)biphenyloxy]-3-hydroxybiphenyl **3** (28 mg, 0.06 mmol), 4'-[2-(*R*)-heptan-2-yloxy]biphenyl-4-carboxylic acid **2**[1] (23.5 mg, 0.072 mmol), EDCI (13.80 mg, 0.072 mmol), DMAP (8.80 mg, 0.072 mmol). Yield: 45.5 mg (94%).

^1H NMR ($CDCl_3$, 400 MHz, δ/ppm): δ 8.26 (dd, 4H, J = 8.42 Hz, 1.83 Hz, biphenyl), 7.65-7.74 (m, 6H, biphenyl), 7.60 (dt, 4H, J = 8.78 Hz, 1.46 Hz, biphenyl), 7.52 (dt, 2H, J = 5.127 Hz, 1.46 Hz, biphenyl), 7.47 (quin, 1H, J = 1.1 Hz, biphenyl), 7.32 (dt, 2H, J = 8.78 Hz, 2.56 Hz, biphenyl), 7.22-7.25 (m, 1H, biphenyl), 7.00 (dd, 4H, J = 8.78 Hz, 2.20 Hz, biphenyl), 4.42 (sxt, 1H, J = 6.22 Hz, $-OC\underline{H}CH_3-$), 4.02 (t, 2H, J = 6.59 Hz, $-OC\underline{H_2}-C_7H_{15}$), 1.73-1.87 (m, 1H+2H, $-OCHCH_3C\underline{H_a}H_b-$, $-OCH_2C\underline{H_2}C_6H_{13}$), 1.57-1.65 (m, 1H, $-OCHCH_3CH_a\underline{H_b}-$), 1.40-1.52 (m, 3H), 1.24-1.40 (m, 18 H), 0.89 (t, 3H+3H, J = 6.95 Hz, $-OC_7H_{14}C\underline{H_3}$, , $-OCHCH_3CH_aH_bC_4H_8C\underline{H_3}$). ^{13}C NMR ($CDCl_3$, 100 MHz, δ/ppm): 165.19, 159.58, 151.43, 150.73, 146.05, 142.94, 142.07, 137.97, 131.93, 131.81, 130.76, 129.84, 128.44, 128.40, 128.32, 127.39, 127.36, 136.61, 124.62, 122.13, 120.65, 120.52, 116.16, 114.97, 74.01, 68.16, 36.46, 31.79, 29.35, 29.28, 29.25, 26.05, 25.54, 22.66, 22.60, 19.75, 14.11, 14.08. Elemental analysis: calculated for $C_{54}H_{58}O_6$: C, 80.77; H, 7.28. Found: C, 80.96; H, 7.36.

Supplementary Note 4. NMR spectra

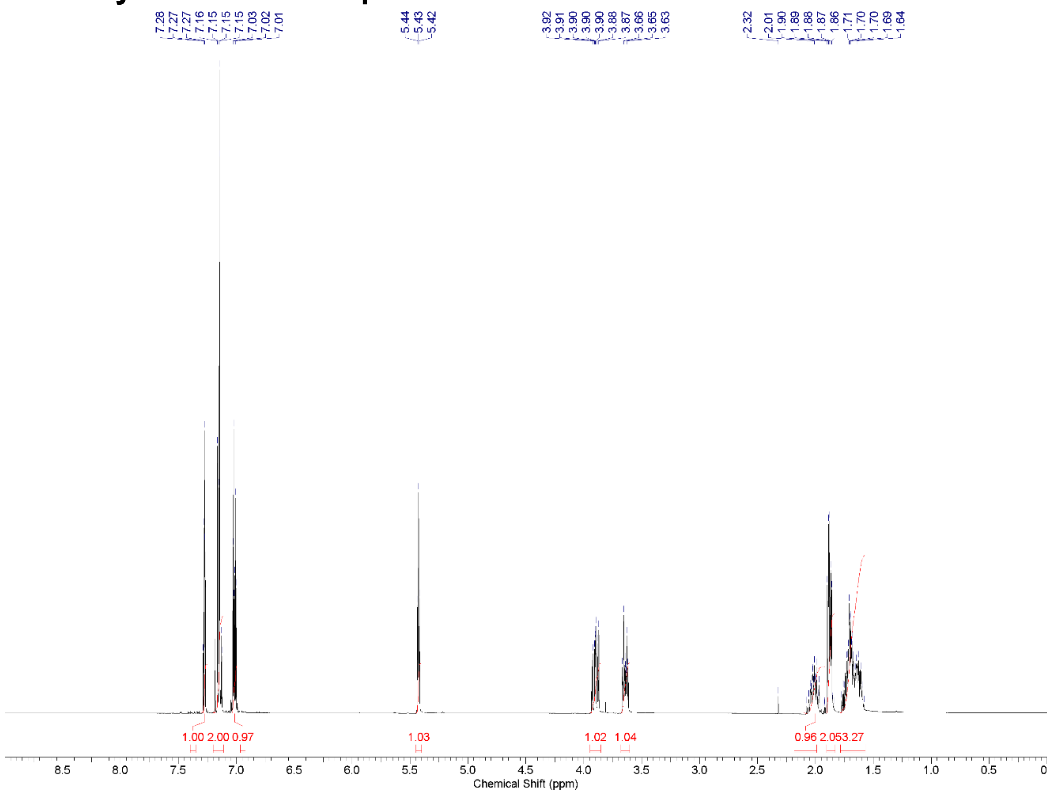

Supplementary Figure 2. ^{1}H NMR spectrum of **7**.

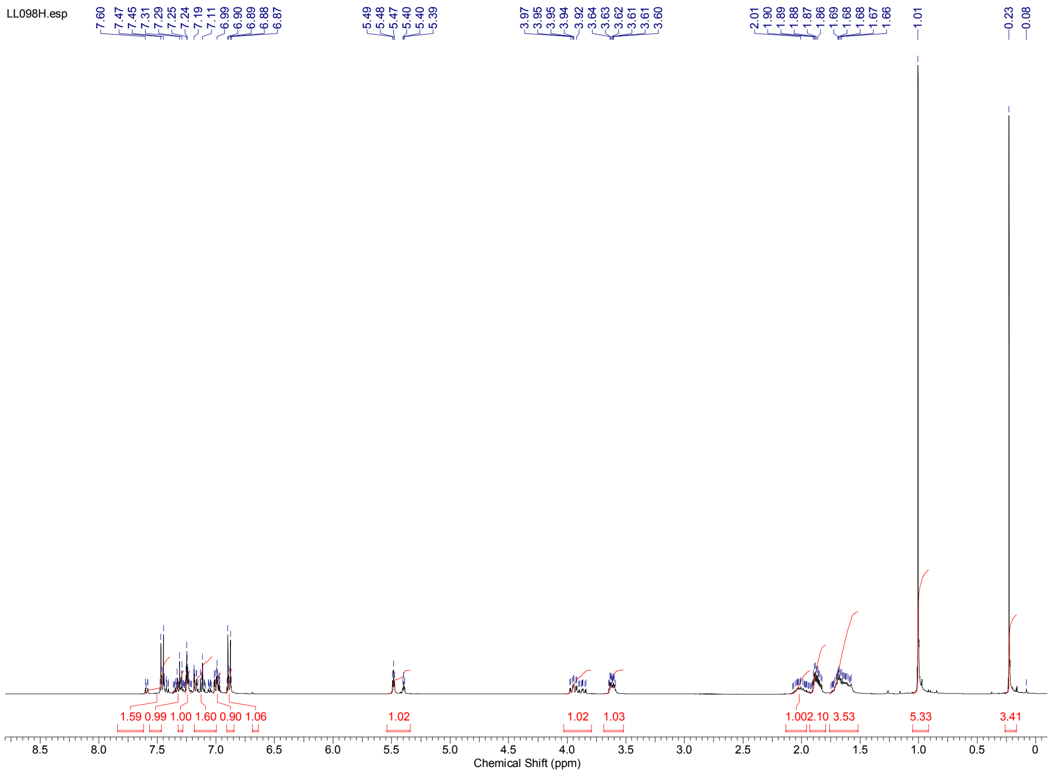

Supplementary Figure 3. ^{1}H NMR spectrum of **6**.

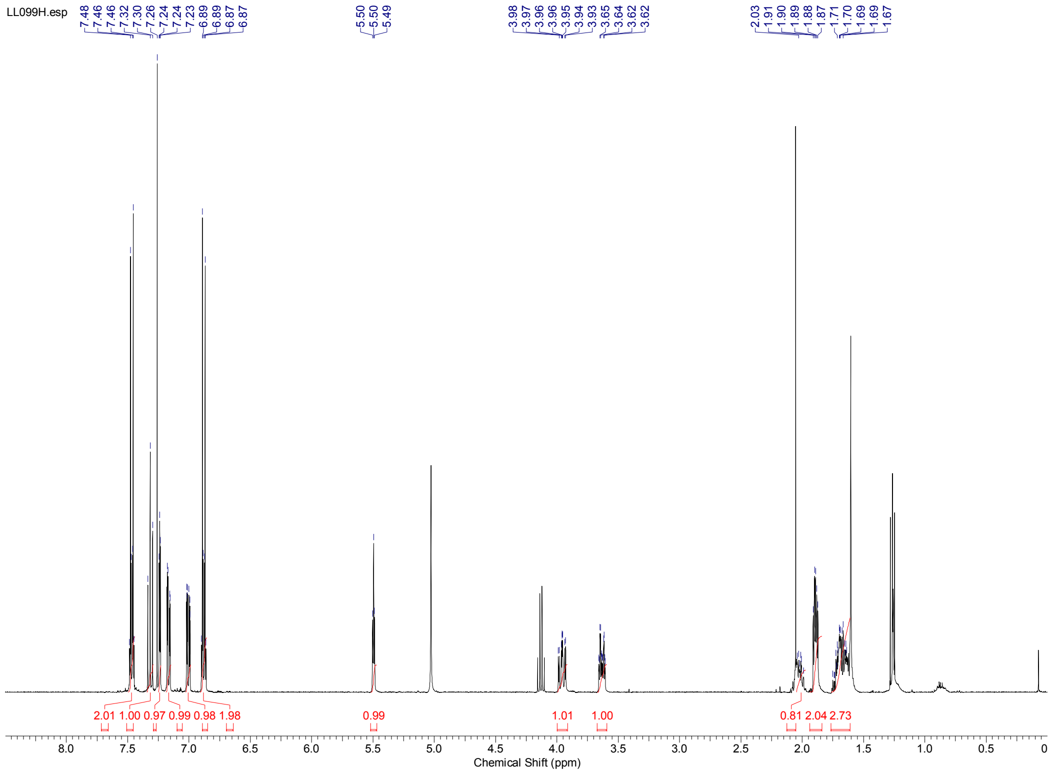

Supplementary Figure 4. ^{1}H NMR spectrum of **5**.

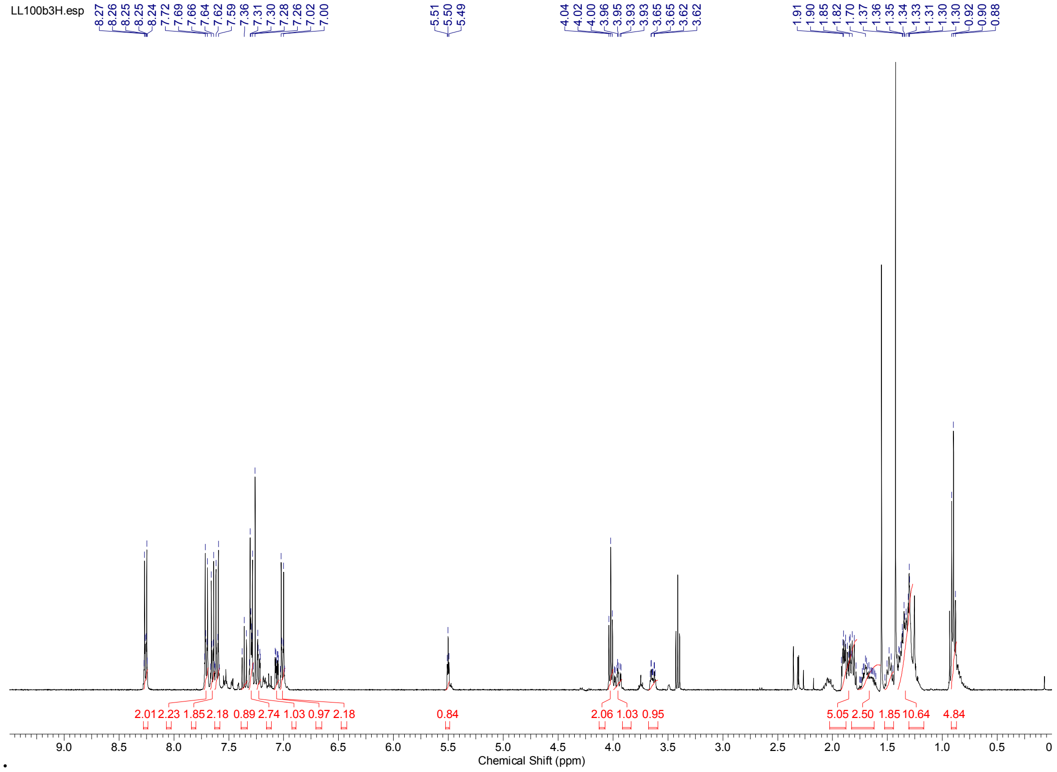

Supplementary Figure 5. ^{1}H NMR spectrum of **4**.

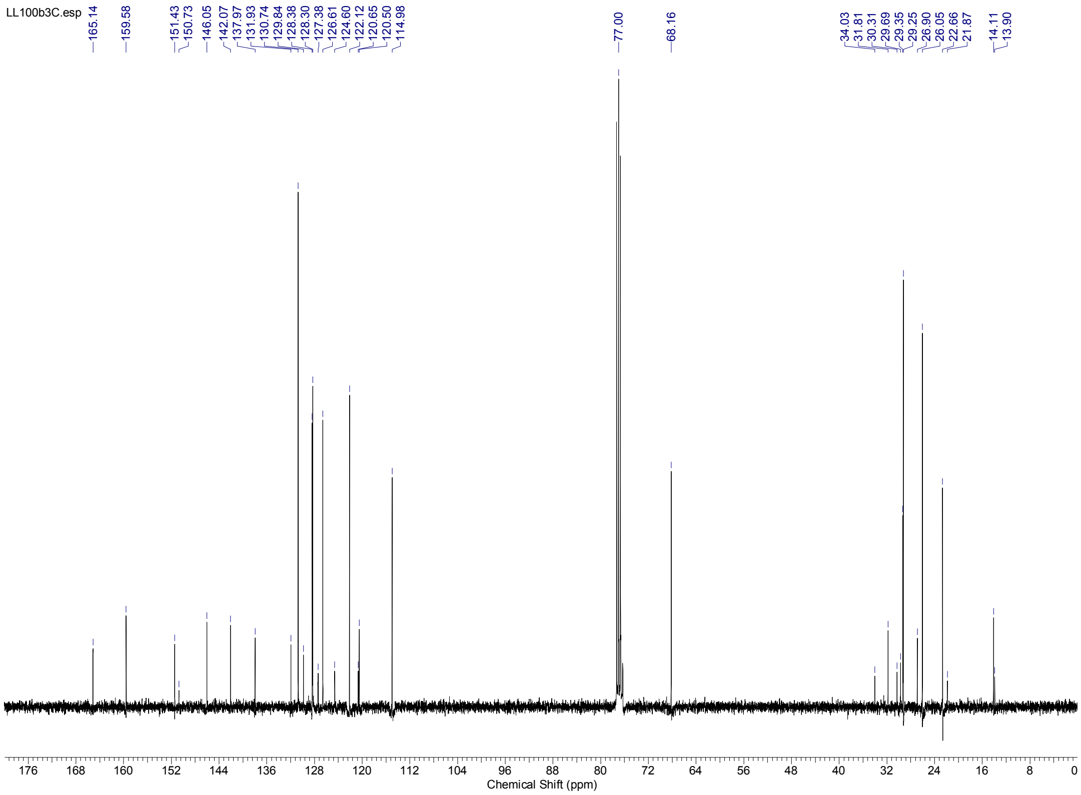

Supplementary Figure 6. ^{13}C NMR spectrum of **4**.

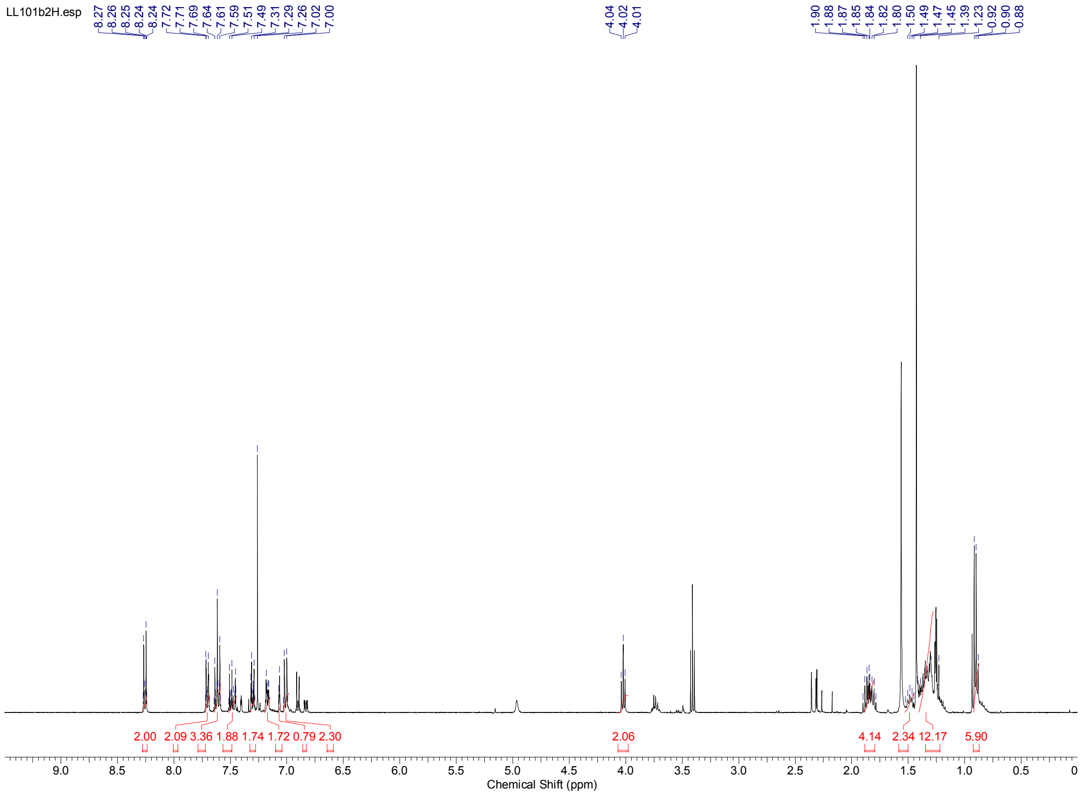

Supplementary Figure 7. ^{1}H NMR spectrum of **3**.

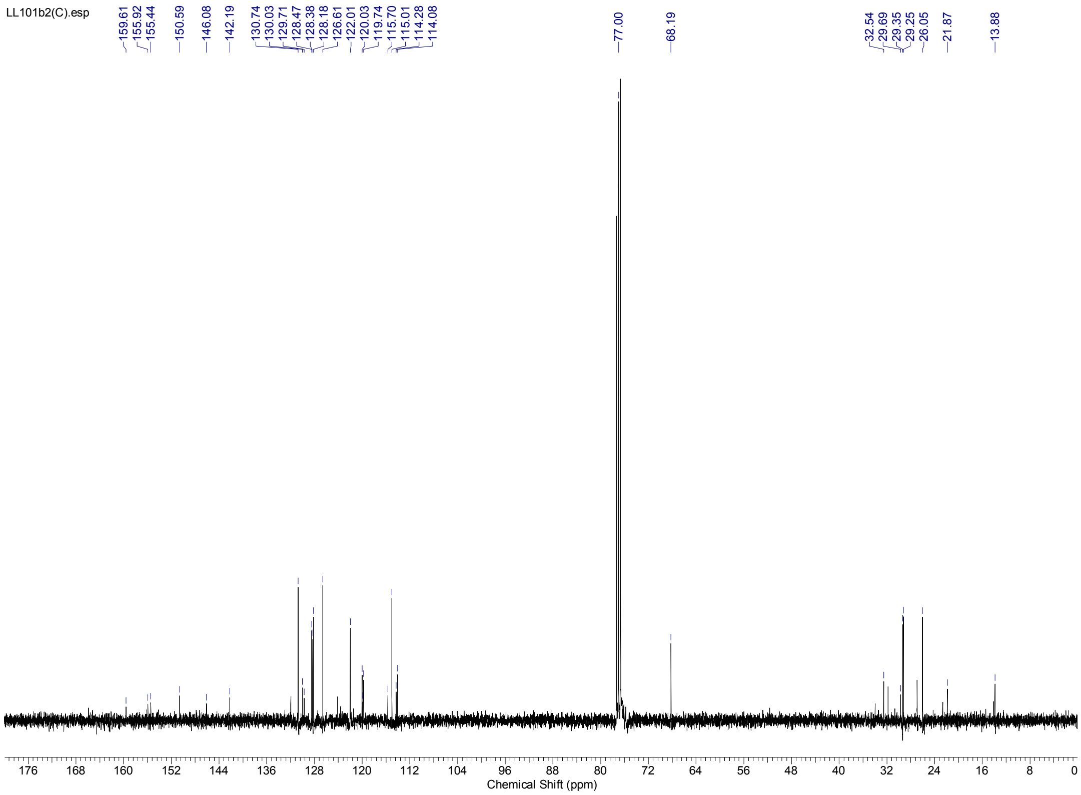

Supplementary Figure 8. ^{13}C NMR spectrum of **3**.

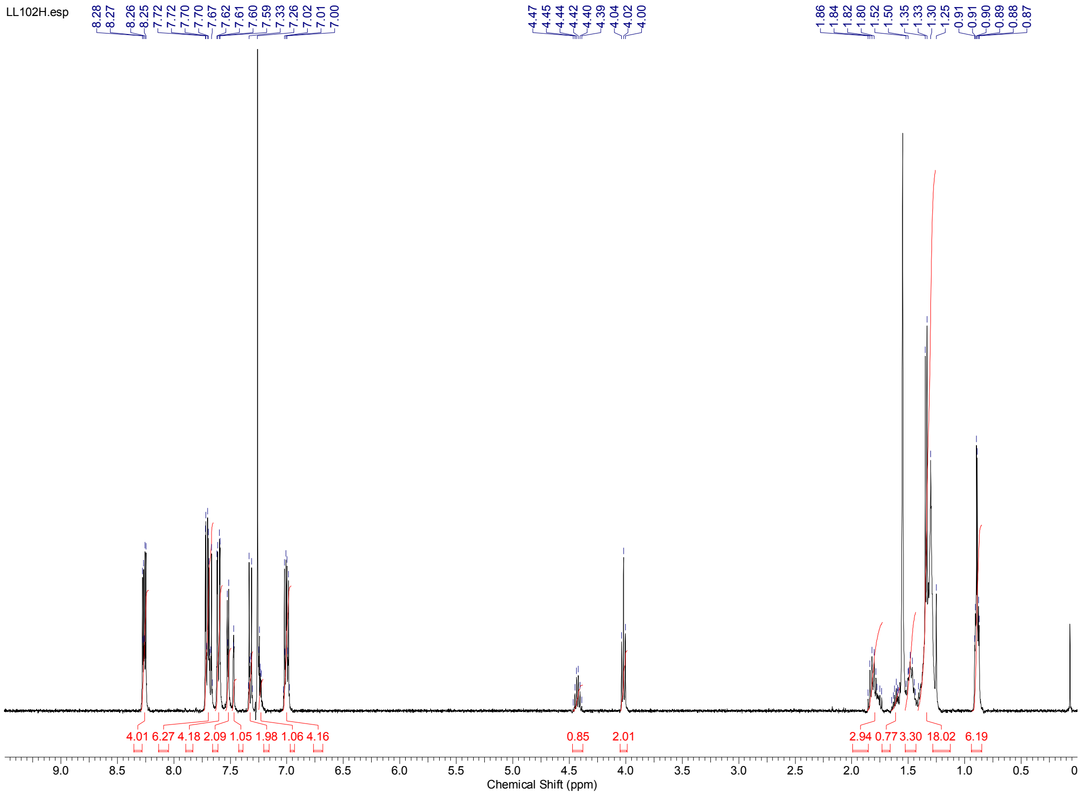

Supplementary Figure 9. ^{1}H NMR spectrum of **1**.

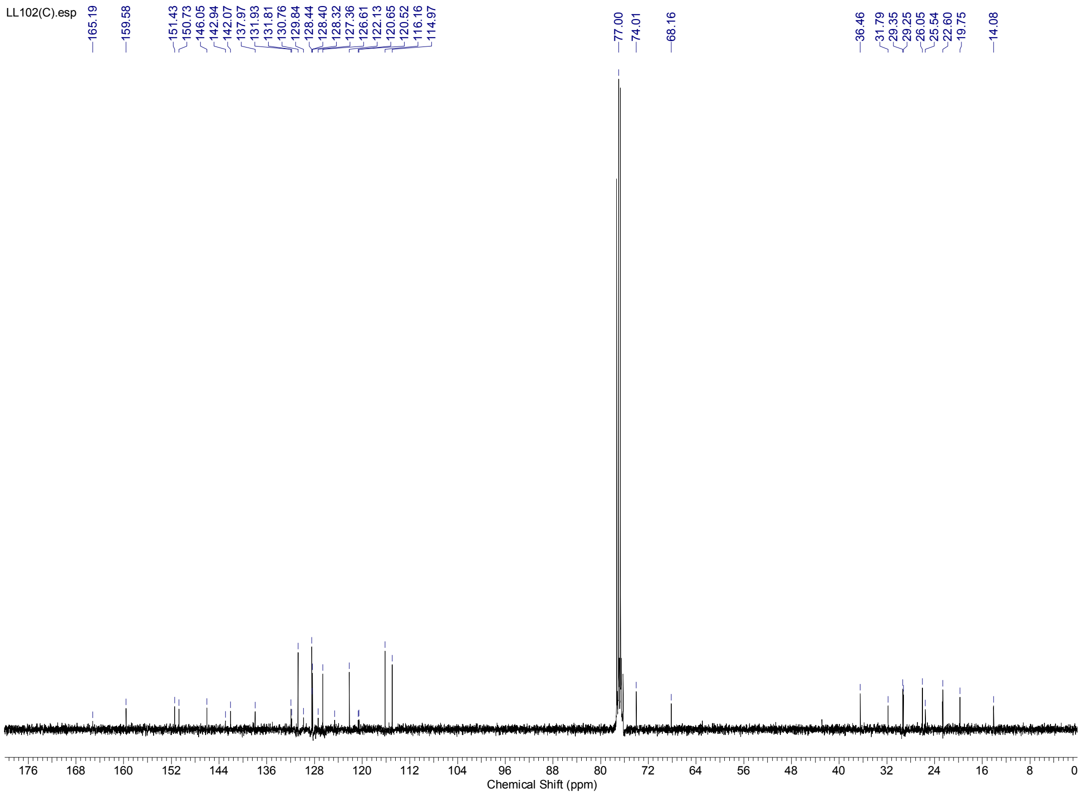

Supplementary Figure 10. ^{13}C NMR spectrum of **1**.

Supplementary Note 5. Additional POM images

5.1 POM – Rapidly cooled sample (sample was obtained by fast cooling from the isotropic liquid phase) on subsequent heating at 2 °C min^{-1}:

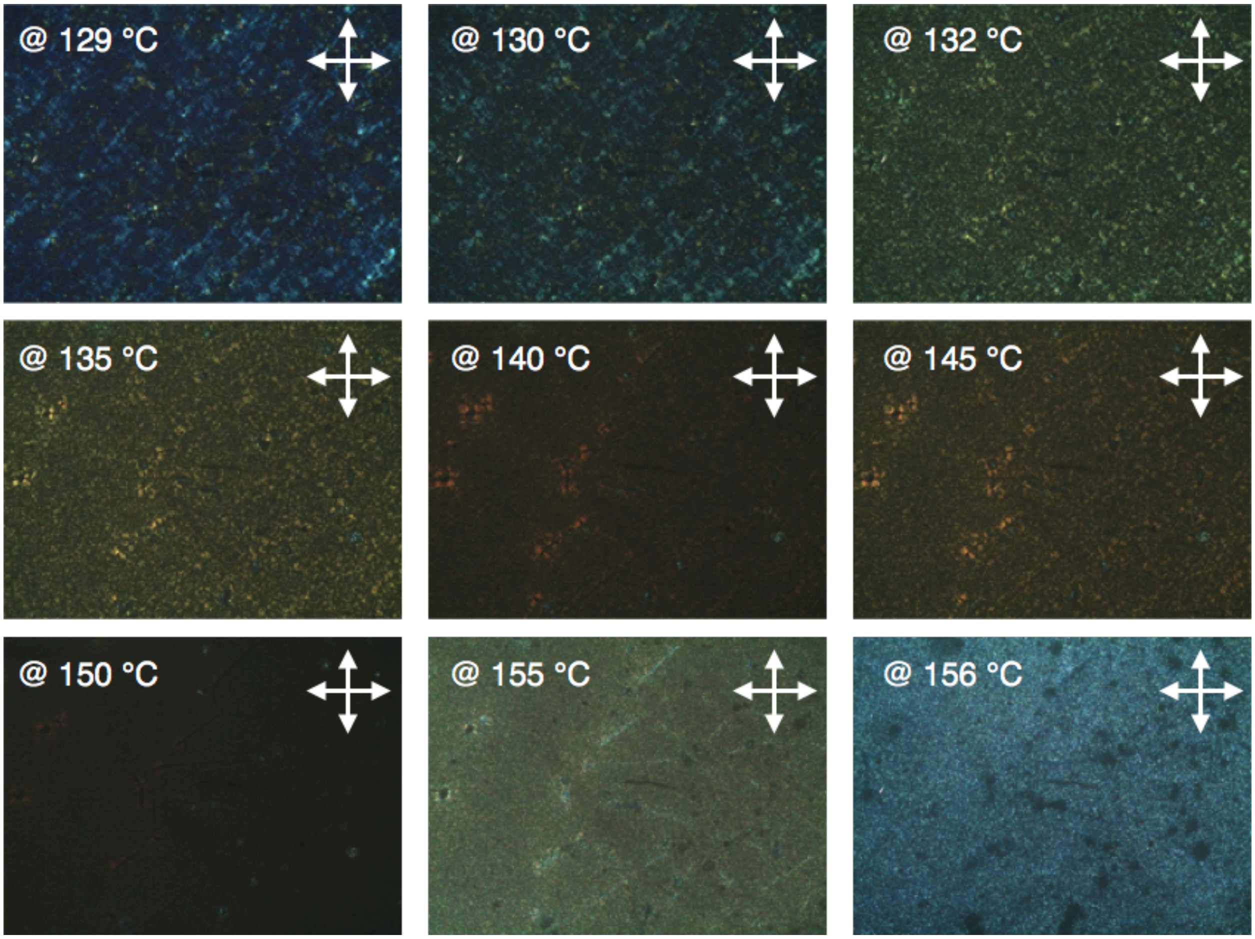

Supplementary Figure 11. Polarized light optical photomicrographs (crossed polarizers) of the rapidly cooled (from the isotropic liquid phase) sample of **1** on subsequent slow heating to the isotropic liquid phase. The material forms an isotropic liquid phase at 156.3 °C using the Linkam heating-cooling stage. Notice the progressive change in birefringence color on heating.

5.2 POM – Slow cooled sample at room temperature

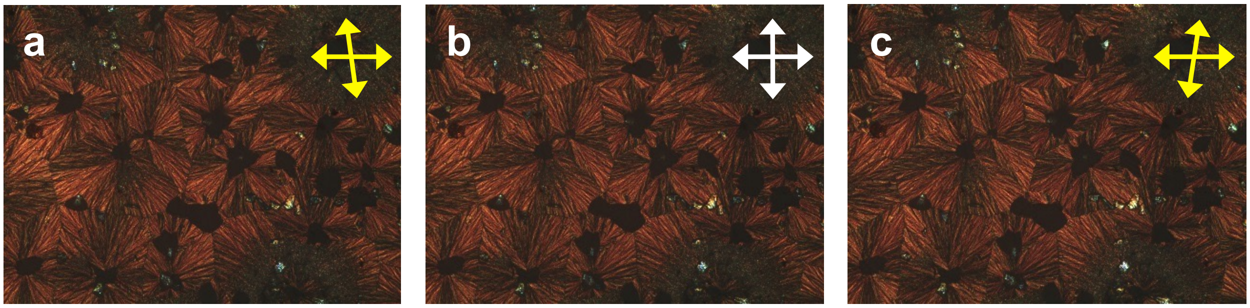

Supplementary Figure 12. Polarized light optical photomicrographs (crossed polarizers) of the slowly cooled (from the isotropic liquid phase) sample of **1** (at 5 °C min^{-1}) at room temperature: **b** with crossed and **a**, **c** with uncrossed polarizers.

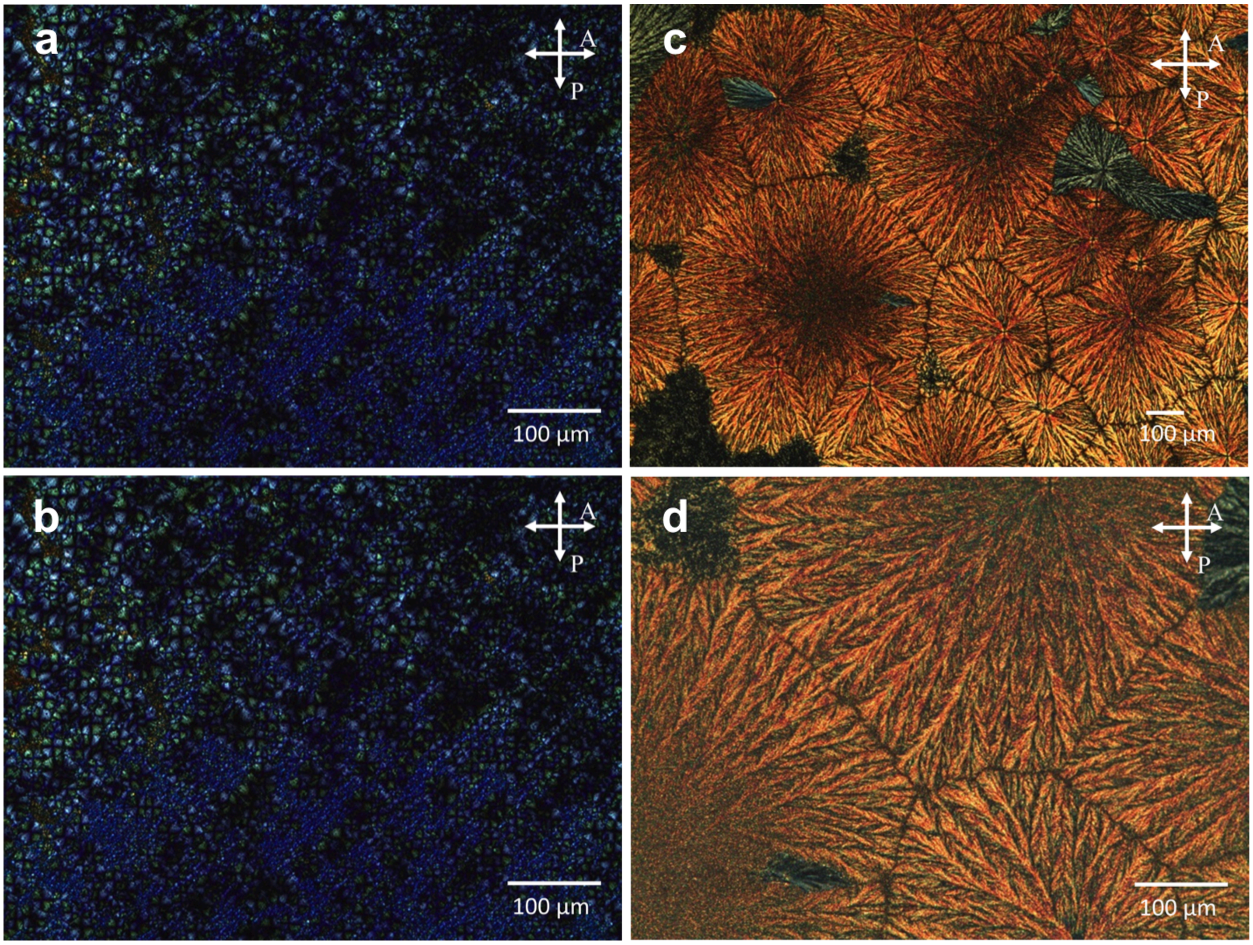

Supplementary Figure 13. Higher resolution and higher magnification polarized optical photomicrographs of compound **1** after heating to the isotropic liquid phase (to about 150 °C). **a**, **b** upon rapid cooling the sample from the isotropic liquid phase; **a** at 130 °C and **b** at 30 °C; **c**, **d** upon slow cooling at a rate of 2 °C min^{-1}: **c** at 142 °C and **d** at 30 °C.

Supplementary Note 6. DSC data

Supplementary Table 1. Collected DSC data for compound **1**.

**Original sample status**[a]	**Rate**[b] °C min^{-1}	**Run**[c]	**Transition Temperature [Enthalpy] on Heating** °C[d] [kJ mol^{-1}]	**Transition Temperature [Enthalpy] on Cooling** ° C[d] [kJ mol^{-1}]
Obtained from recrystallization, Plot **a**	5	1st	153.2 [41.51]	140.1 [−38.31]
		2nd	152.8 [40.82]	139.9 [−38.50]
Heated to Iso and then quickly cooled in the air, Plot **b**	5	1st	152.3 [37.71]	137.0 [−35.73]
		2nd	152.3 [37.47]	136.9 [−35.94]
Heated to Iso phase then cooled at 5 °C min^{-1}, Plot **c**	50	1st	154.3 [38.86]	125.9 [−33.52]
		2nd	154.3 [36.04]	128.4 [−33.67]

[a] Treatment of the sample prior to DSC measurements, [b] heating/cooling rate set for the DSC experiment, [c] 2nd heating/cooling run in the DSC heater, [d] peak values are used.

a

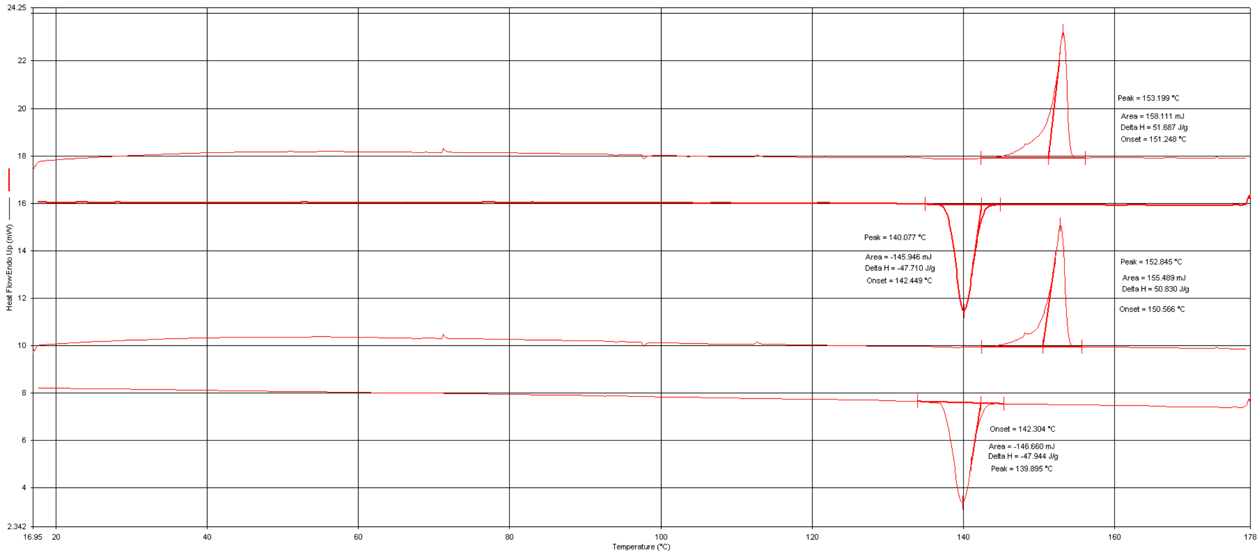

b

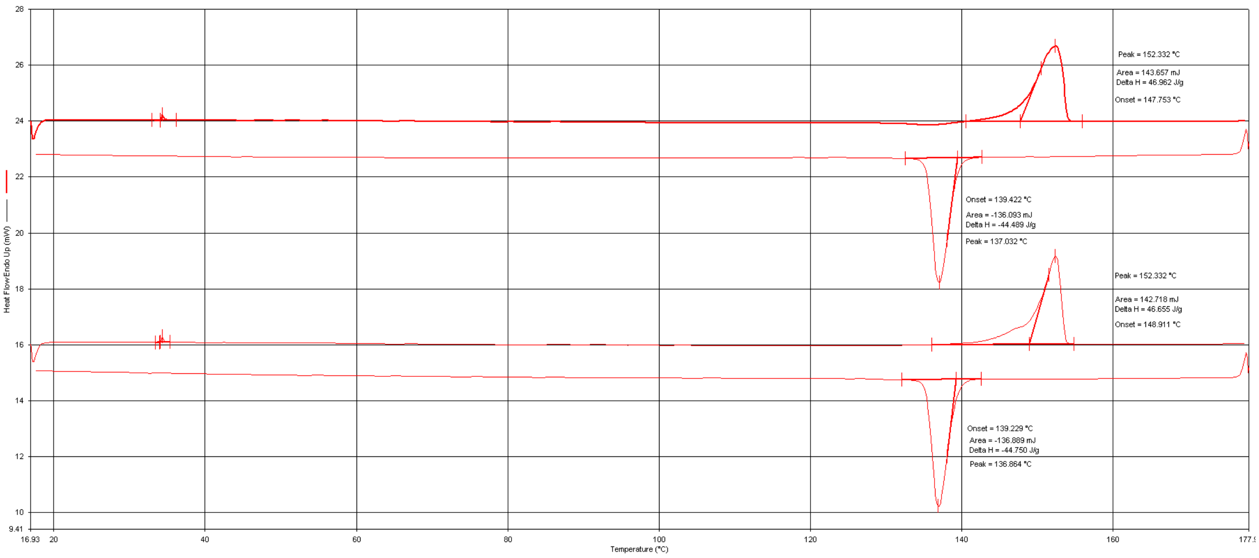

c

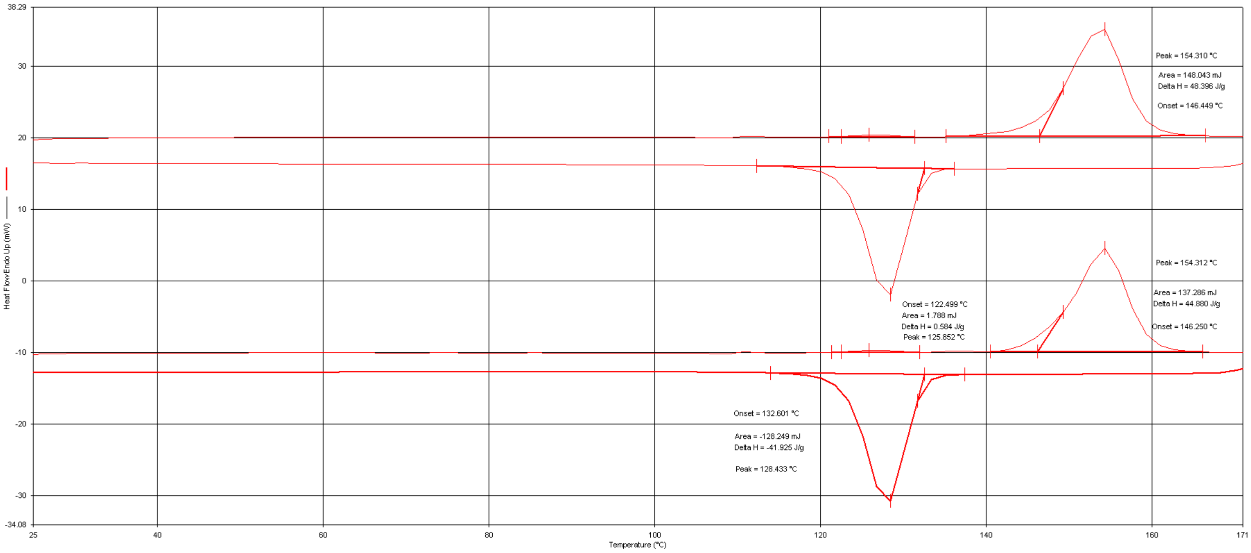

Supplementary Figure 14. DSC plots for compound **1** directly taken from the Perkin Elmer Pyris 1 software interface. **a** Sample was obtained directly from recrystallization and measured at 5 °C min^{-1}. In this case, the sample forms the B1 (*p*2/*m*, Col_{ob}) phase based on POM observations. The phase formed is monotropic (i.e. only observed on cooling). **b** Sample was obtained by heating to the isotropic liquid phase and then thermally quenched (outside of the DSC instrument) and then measured at a rate of 5 °C min^{-1} by DSC. In this case, the sample initially forms the HµF B4 phase on first heating. **c** Sample was obtained by cooling from the isotropic liquid phase at 5 °C min^{-1} (outside of the DSC instrument) and then measured by DSC at a rate of 50 °C min^{-1}. In this case, the sample initially forms the B1 (*p*2/*m*, Col_{ob}) phase, which should then coexist with the HµF B4 phase during the 50 °C min^{-1} cooling runs. It appears that the broader phase transitions occurring at lower temperatures are a consequence of the 10-fold higher heating/cooling rate and phase coexistence.

Supplementary Note 7. Additional CD spectra

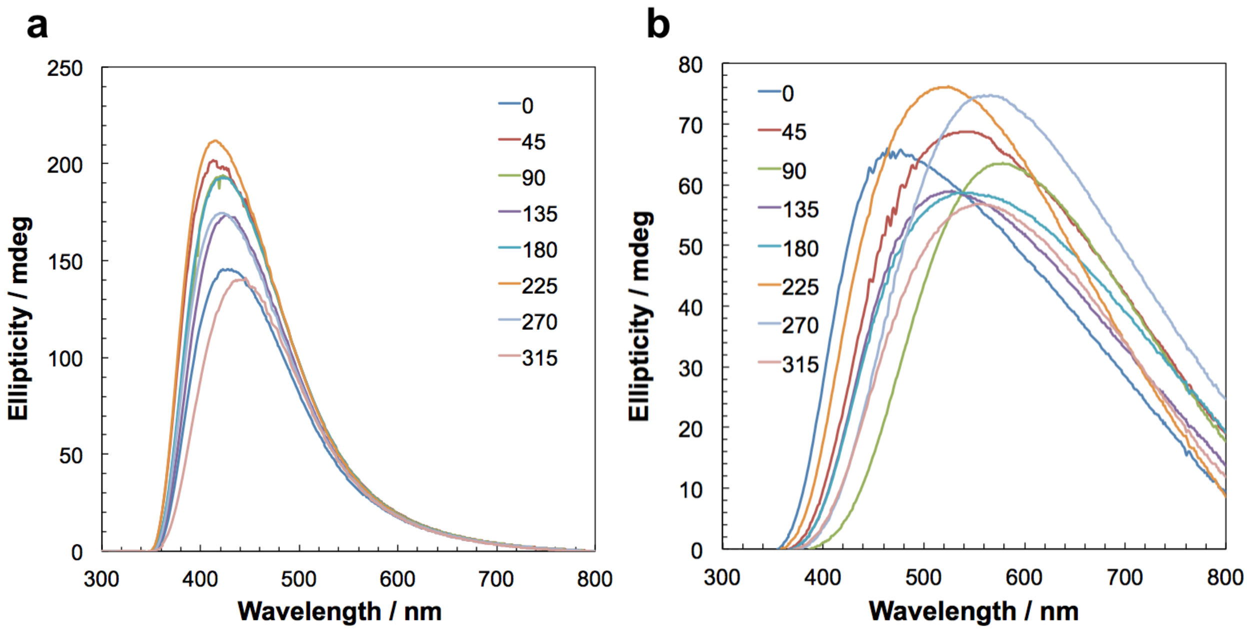

Supplementary Figure 15. CD spectra of thin LC films between untreated quartz substrates of compound **1** on cooling at different cooling rates from the isotropic liquid phase at different sample rotation angles as indicated in each legend: **a** rapid cooling and **b** slow cooling at a rate of 2 °C min^{-1}.

Supplementary Note 8. Reflection and transmission UV-vis spectra

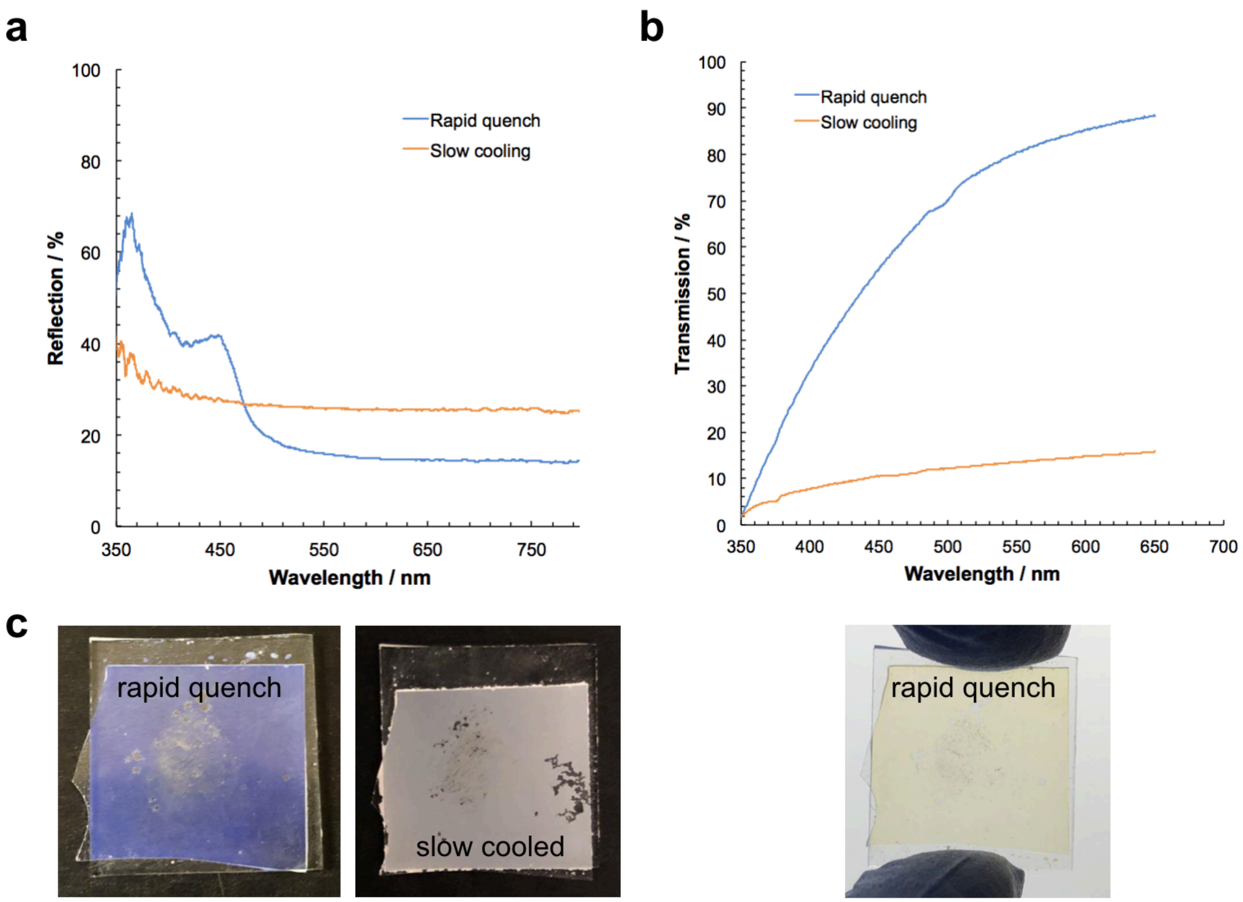

Supplementary Figure 16. **a** UV-vis reflection and **b** UV-vis transmission spectra of the slow and fast cooled sample of **1**, respectively (blue curves: fast cooled; orange curves: slow cooled). **c** Sample photographs in reflection and transmission. In transmission, the slow cooled sample appears very similar to the fast cooled sample.

Supplementary Note 9. Comparison B4 and B7 phase and types of B1

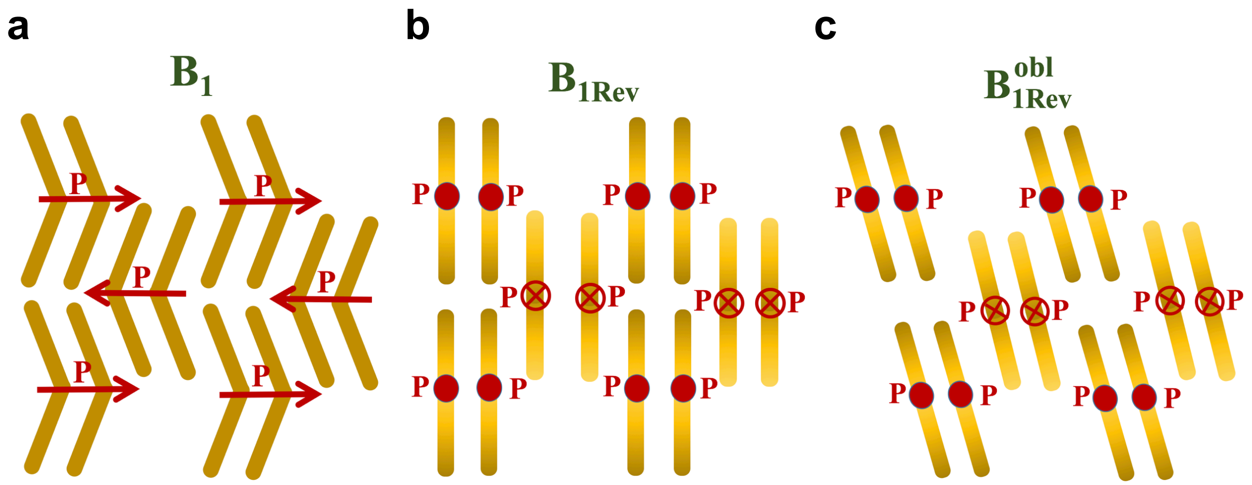

Supplementary Figure 17. Schematic representation of the three main known columnar phases formed by bent-core molecules.[23] **a** B_1 phase with polar smectic ribbons with positions shifted periodically by half molecular lengths and polarizations alternating left and right normal to the ribbons; **b** B_{1Rev} phase with polar smectic ribbons with positions shifted periodically by half molecular lengths and polarizations alternating in and out along the ribbons; **c** $B^{obl}{}_{1Rev}$ phase with tilted polar smectic ribbons with positions shifted periodically by half molecular lengths and polarizations alternating in and out along the ribbons.

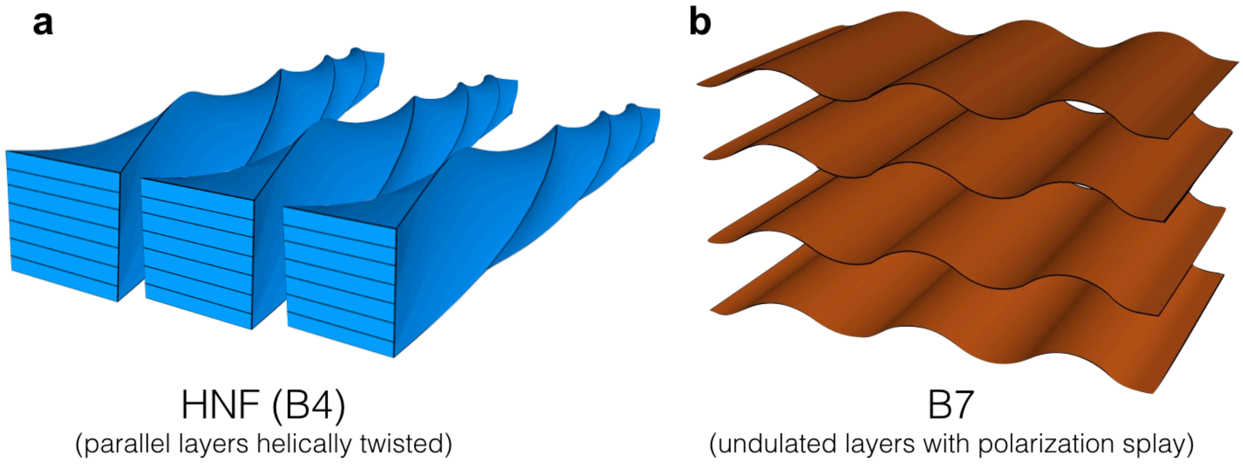

Supplementary Figure 18. A comparison between models of: **a** the B4 HNF and **b** the B7 phase. The B7 phase is characterized by one-dimensional layer undulations and periodic polarization splay.[2]

Supplementary Note 10. Additional SAXS data

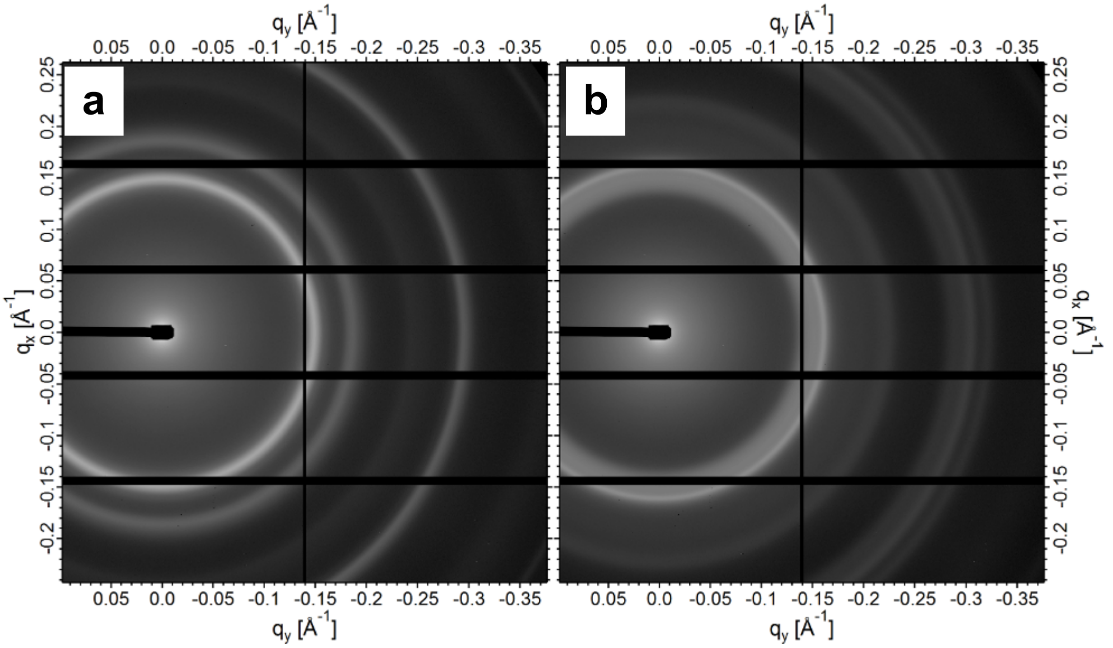

Supplementary Figure 19. 2D SAXS patterns of **1**: **a** on slow cooling at 5 °C min^{-1} and **b** on fast cooling.

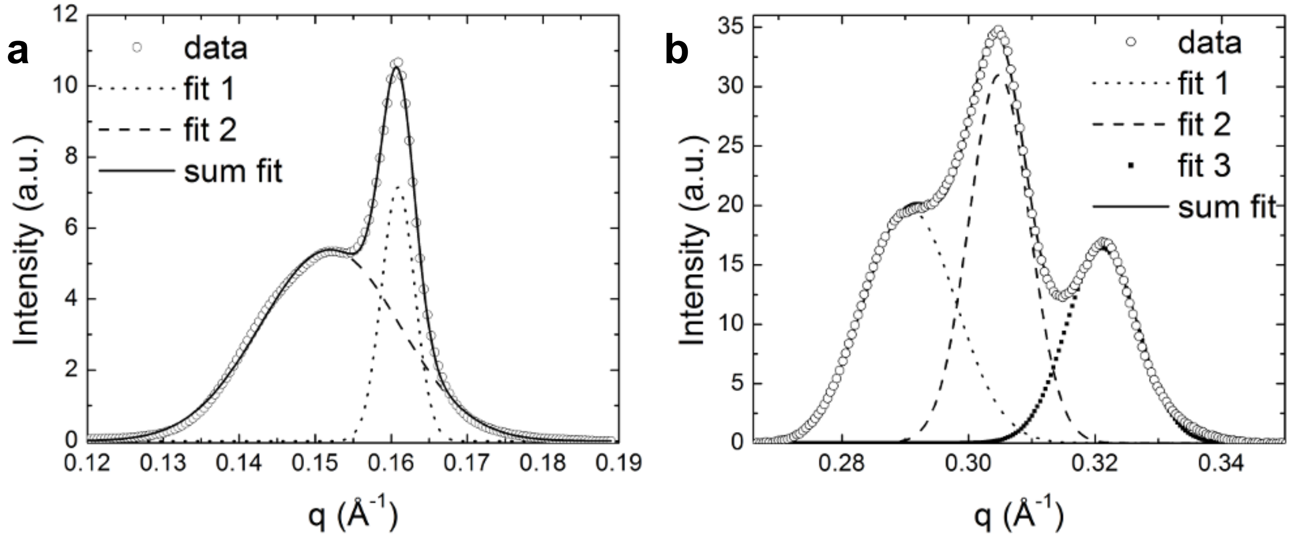

Supplementary Figure 20. A fit of: **a** * and $q^Q{}_1$, and **b** $q^Q{}_3$, *, and $q^Q{}_4$ from SAXS data of the rapidly cooled sample of compound **1**.

Supplementary Note 11. Additional SEM images

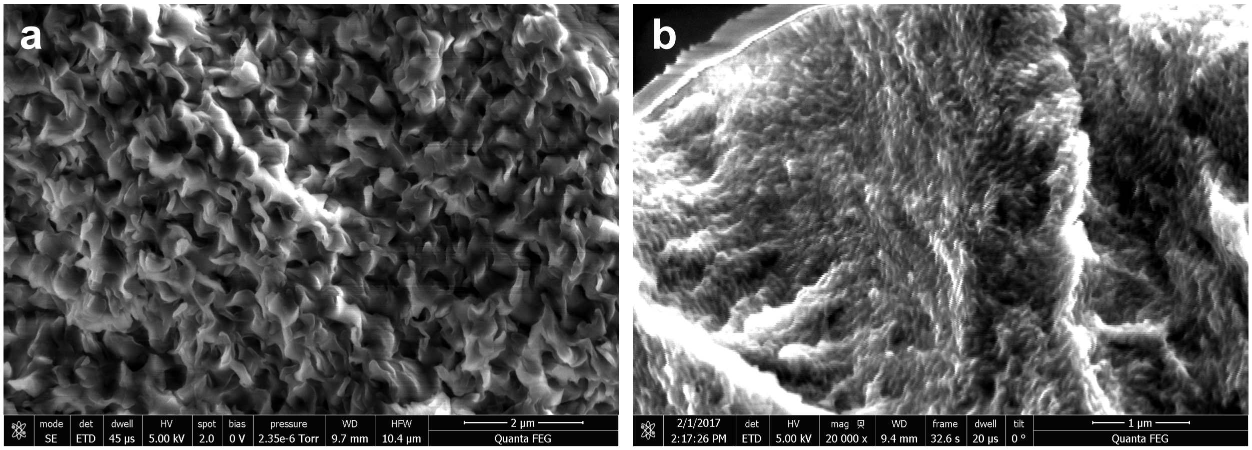

Supplementary Figure 21. SEM images of: **a** compound **1** after rapid cooling from the isotropic liquid state (scale bar 2 µm) and **b** compound **II** (scale bar 1 µm). The feature size differences between filaments of the HNF_{mod2} (pitch: ~200 nm and width: ~40 nm) formed by **II** and the new HµF phase formed by **1** are clearly visible. No Au was deposited on the sample prior to imaging.

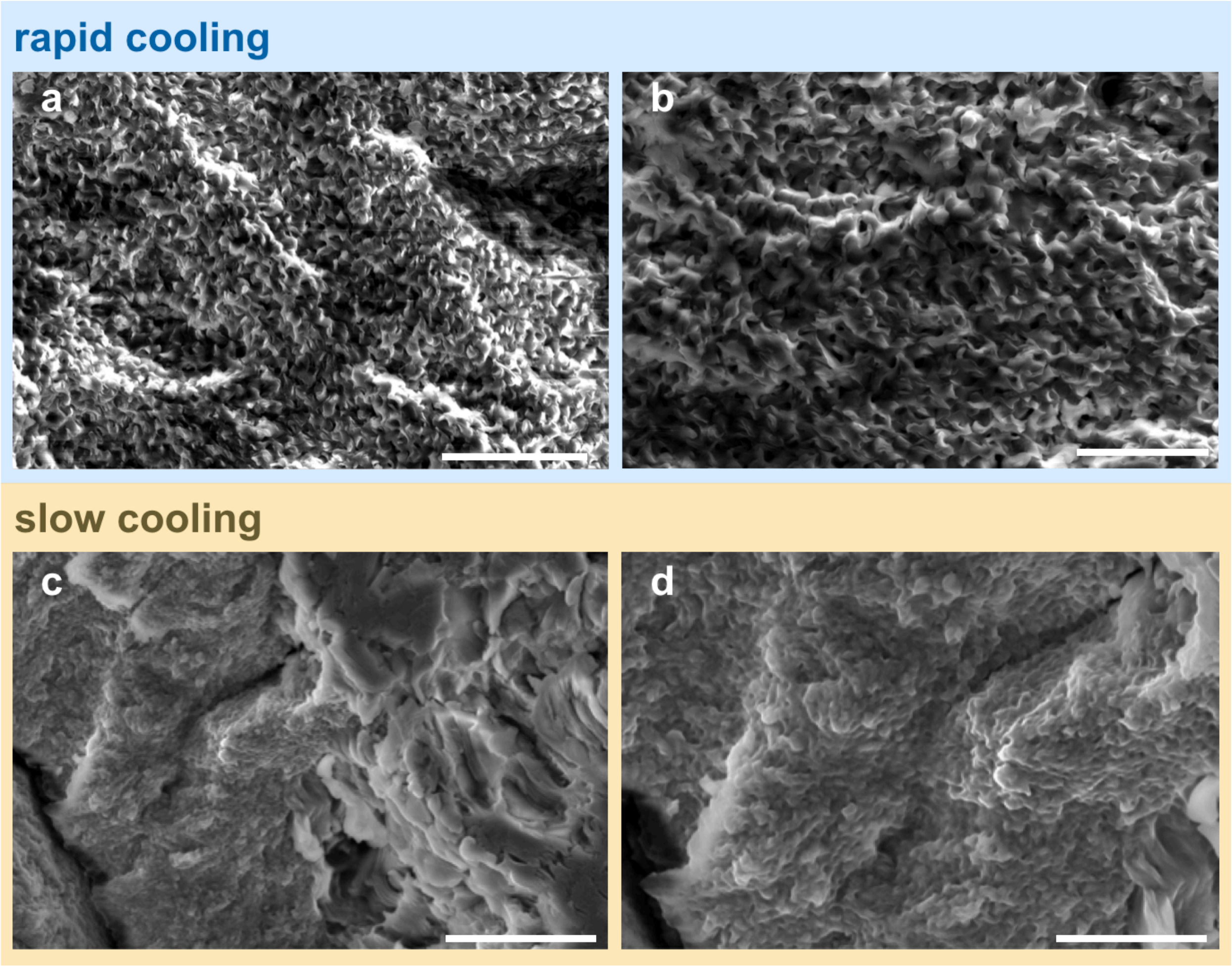

Supplementary Figure 22. Additional SEM images of the phase of **1** obtained: **a**, **b** on quenching from the isotropic liquid state to room temperature at two different magnifications of the same sample and **c**, **d** on slow cooling at a rate of 5 °C min^{-1} from the isotropic liquid phase to room temperature. Each sample (thickness about 200 μm) was imaged directly; i.e. no metal was deposited prior to imaging. Scale bars: **a** 5 μm, **b** 3 μm, **c** 4 μm, and **d** 2 μm.

Supplementary Note 12. Contact preparations between 1 and II, 1 and III as well as II and III

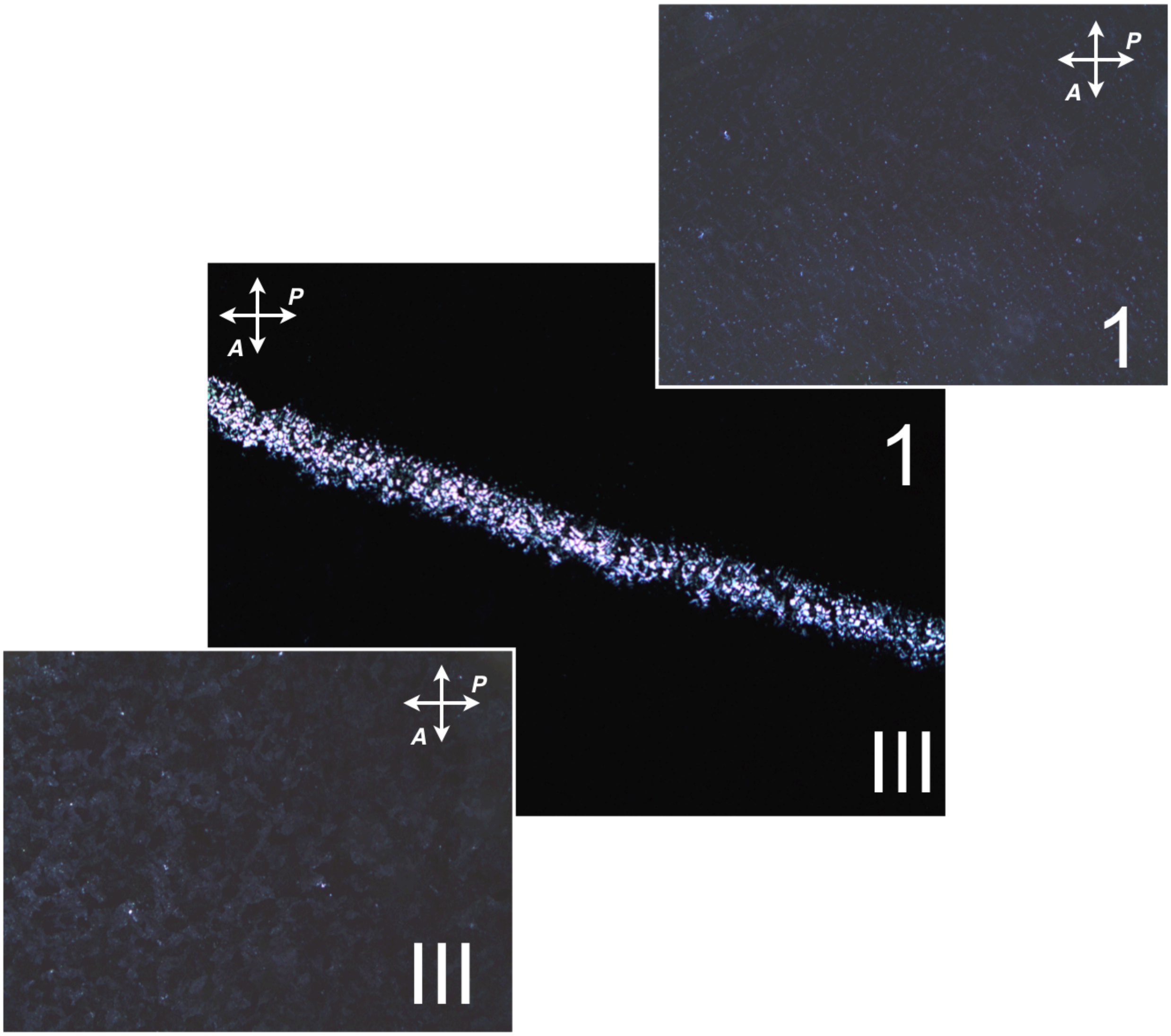

Supplementary Figure 23. Contact preparation between **1** and **III** upon rapid cooling from the isotropic liquid phase of both compounds. Since the contact region shows an induced more birefringent phase, separate images of the two areas above and below this contact region with more appropriate contrast are shown as well.

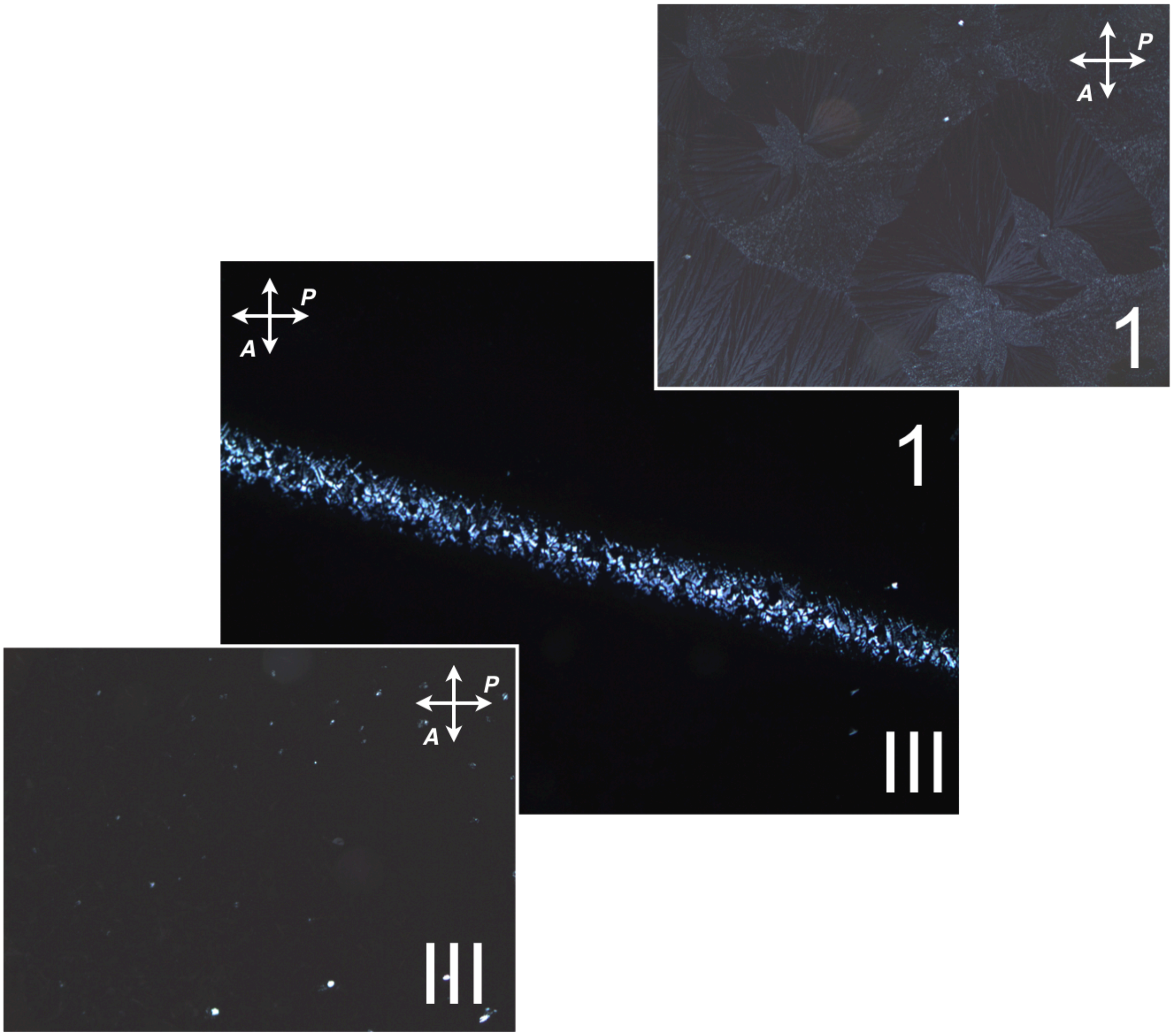

Supplementary Figure 24. Contact preparation between **1** and **III** upon slow cooling from the isotropic liquid phase of both compounds at a rate of 2 °C min^{-1}. Since the contact region shows an induced more birefringent phase, separate images of the two areas above and below this contact region with more appropriate contrast are shown as well.

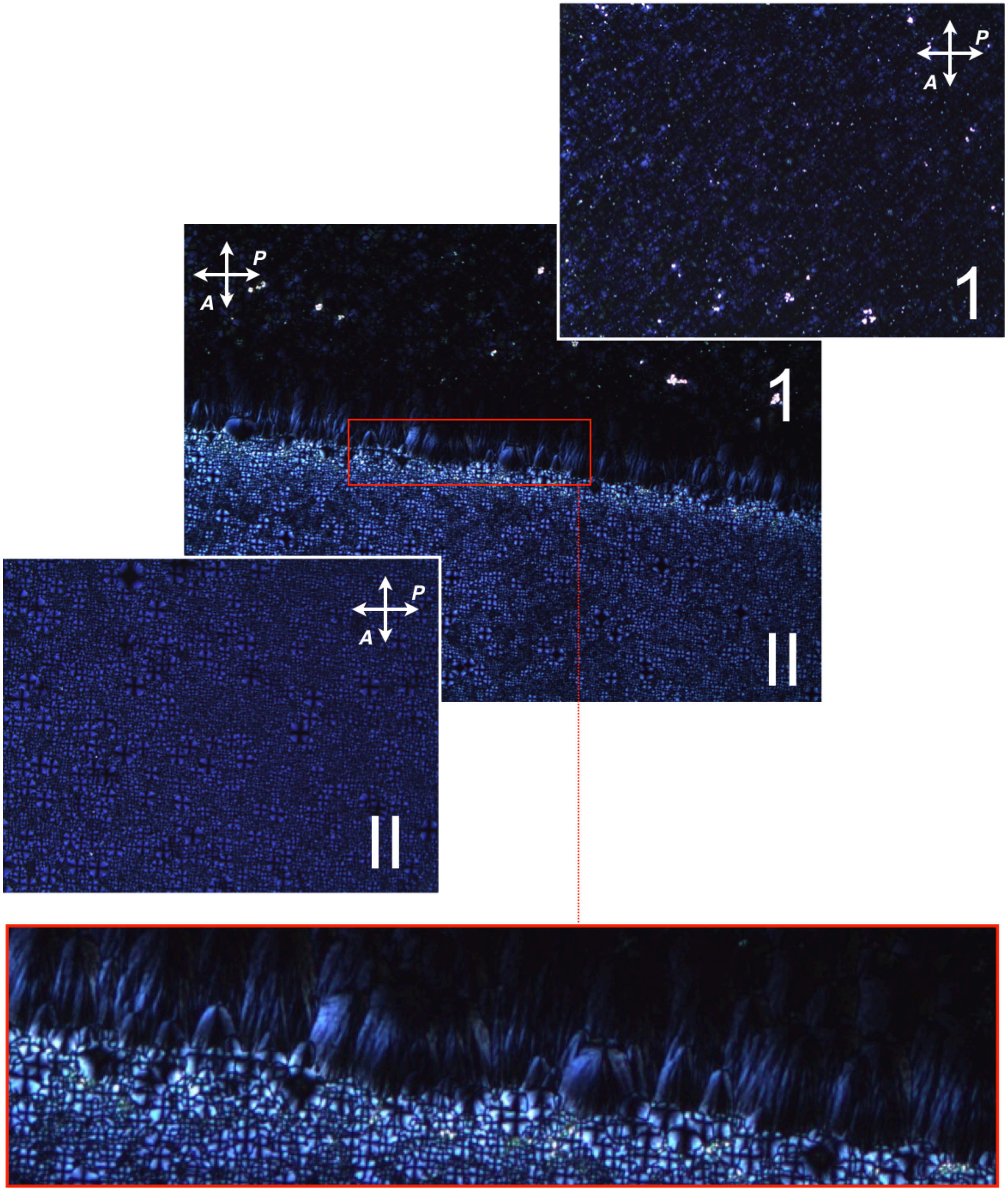

Supplementary Figure 25. Contact preparation between **1** and **II** upon rapid cooling from the isotropic liquid phase of both compounds. Separate images of the two areas above and below the contact region with more appropriate contrast are shown as well. In addition, the red-framed image at the bottom shows a magnified area of the contact zone.

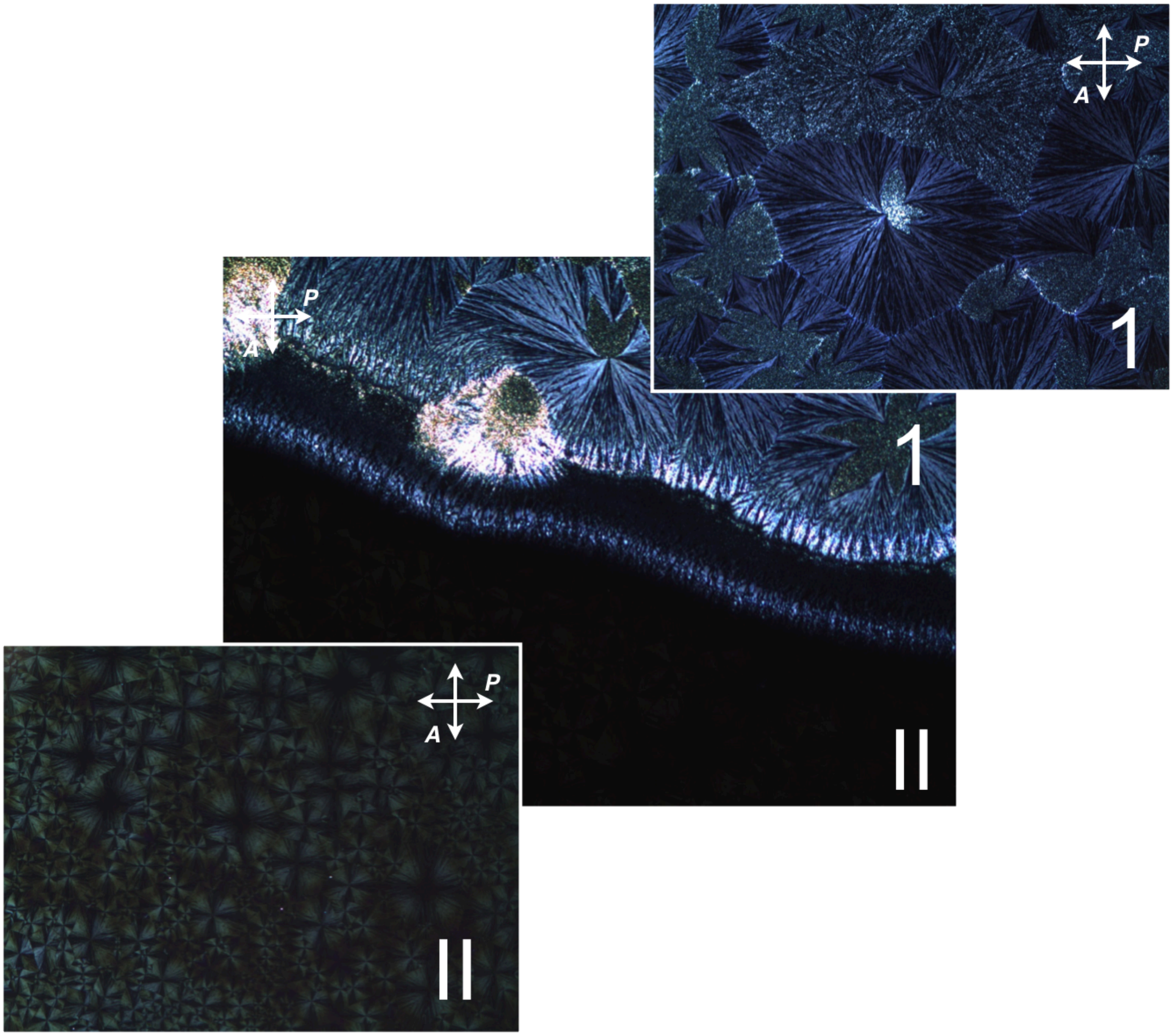

Supplementary Figure 26. Contact preparation between **1** and **III** upon slow cooling from the isotropic liquid phase of both compounds at a rate of 2 °C min^{-1}. Separate images of the two areas above and below this contact region with more appropriate contrast are shown as well.

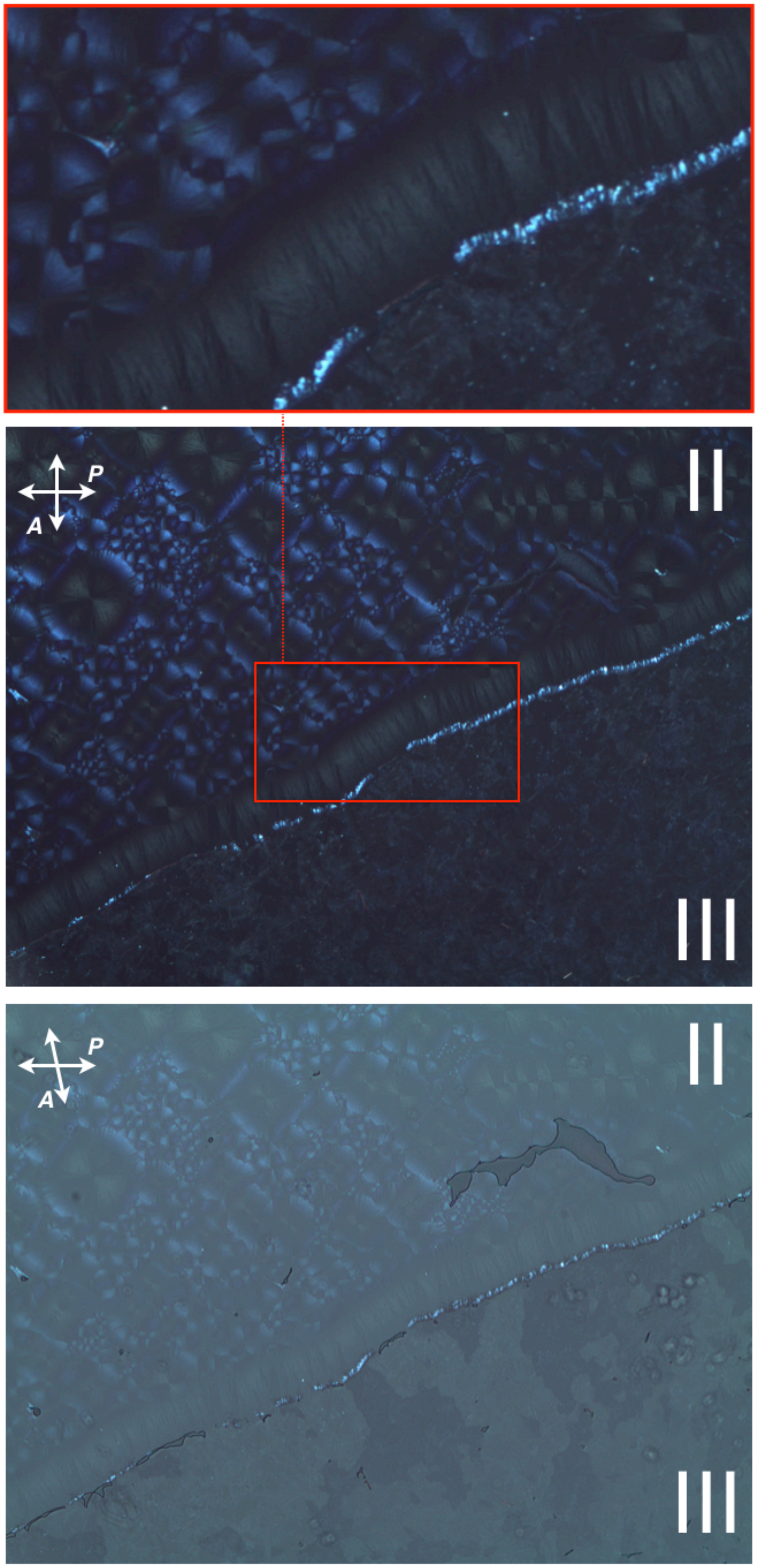

Supplementary Figure 27. Contact preparation between **II** and **III** upon slow cooling from the isotropic liquid phase of both compounds at a rate of 5 °C min^{-1}: (top) with crossed and (bottom) uncrossed polarizers (clearly indicating the chiral HNF domains of the achiral compound **III**).

Supplementary Note 13. Comparison of HNF, HNF_{mod}, HNF_{mod2}, B7 and B1 phases

HNFs are enormously attractive for photovoltaic as well as chiral separation applications and as templates for the chiral spatial assembly of guest molecules.[3, 4, 5] HNF phases of bent-core LCs form as the result of an intra-layer mismatch between top and bottom molecular halves that is relieved by local saddle-splay geometry.[6, 7, 8] The phase consists of bundles of approximately 7 twisted smectic layers 20 to 50 nm in width featuring a helical pitch of ~200 nm. Solid-state NMR data confirmed in-plane crystalline order, where a competition between the smectic layering and conformational chirality induces twisted filaments separated by chirality.[9, 10] Macroscopically the phase is racemic (a conglomerate of left and right-handed homochiral domains) when achiral molecules are used. Chiral side chains[11, 12] and chiral memory (*i.e.* temperature cycling to an adjacent chiral SmCP phase or B2 texture[13]) produce homochiral HNF phases. On the contrary, the addition of a chiral additive (dopant) in most cases leads to the formation of diastereomeric systems with equal amounts of left and right-handed HNF nucleation sites that serve as host for the chiral additive.[14, 15] A secondary twist, meaning stacked arrays of nanofilaments are not parallel, but rotated with respect to each other, is responsible for both the commonly observed blue structural color of most HNF materials and their ambidextrous optical activity.[16] Confinement of HNF phases in nanoporous anodic aluminum oxides or nanobowls can be used to tune the morphology including the helical pitch, the layer spacing, and the number of layers of confined single HNFs by adjusting the pore diameter of the confining nanopores.[5, 17, 18, 19, 20, 21] In addition, tuning the molecular structure led to the discovery of modulated (HNF_{mod}) conglomerates[22] and dual modulated homochiral HNF phases (HNF_{mod2} featuring homochiral HNFs with a twist sense matching the secondary twist) with additional in-layer as well as interlayer electron density modulations.[1] The latter dual modulation of the HNF_{mod2} phase, formed by compound **II** (Figure 1) as well as the analogue material with opposite chirality in each side chain suggests a columnar local structure within the nanofilaments (with lattice parameters of a = 43.4 Å and b = 38.0 Å) and may represent a combination of the two phases formed by the parent achiral compound **III** (Col_r and HNF).[1]

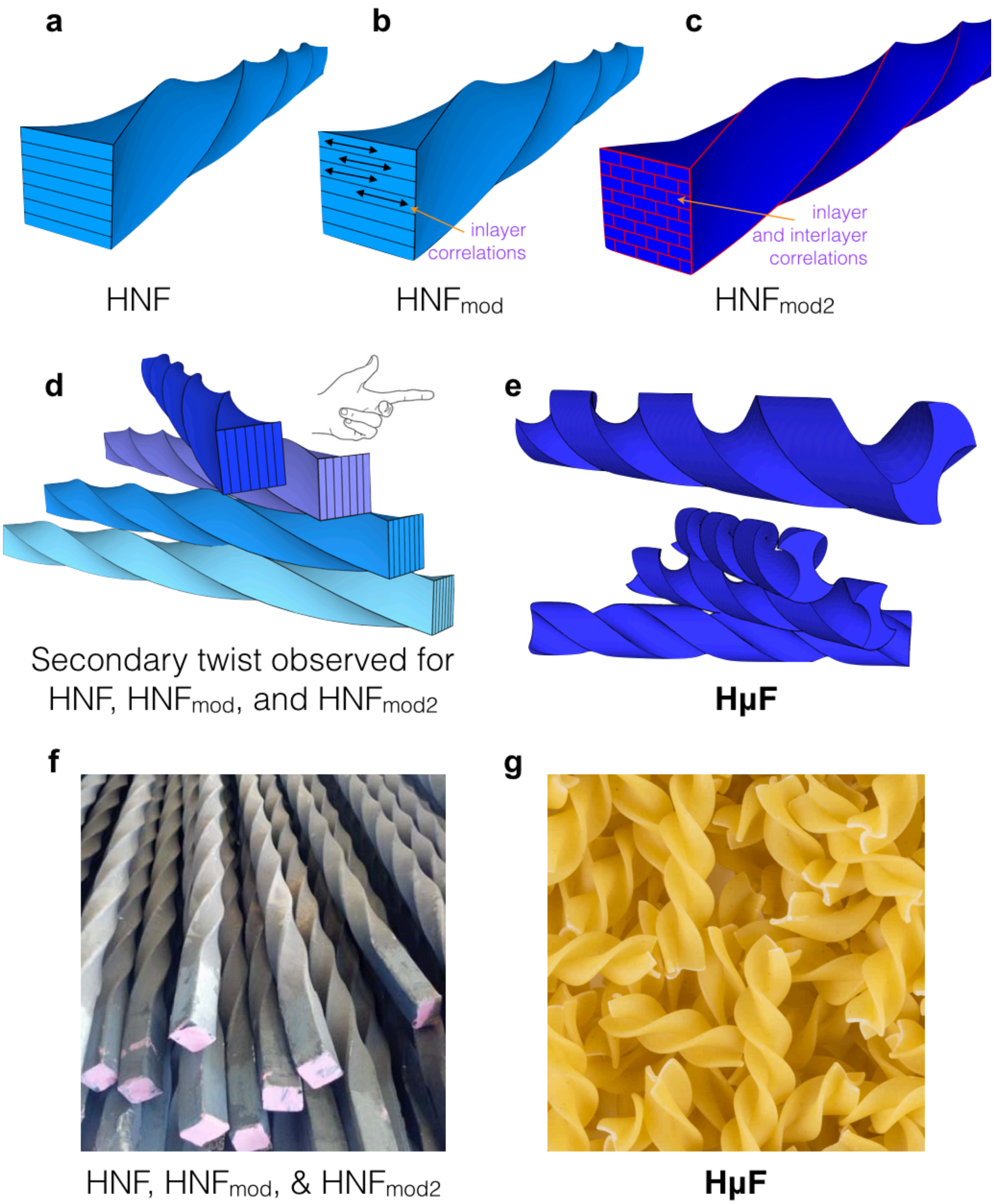

Supplementary Figure 28. Models for HNF phases: **a** HNF, **b** HNF_{mod}, and **c** HNF_{mod2}. **d** An example of a left-handed secondary twist as observed for HNF, HNF_{mod} and HNF_{mod2} phases. **e** Corkscrew or drill-like structure of the right-handed filaments formed by **1** upon rapid cooling together with the suggested secondary right-handed twist. **f** and **g** show examples of twisted square metal rods for HNF, HNF_{mod} and HNF_{mod2} phases as well as rotini noodles as an example for the HμF phase.

Supplementary References

1. Li L, Salamonczyk M, Jákli A, Hegmann T. A dual modulated homochiral helical nanofilament phase with local columnar ordering formed by bent core liquid crystals: effects of molecular chirality. *Small* **12**, 3944-3955 (2016).
2. Coleman DA*, et al.* Polarization-modulated smectic liquid crystal phases. *Science* **301**, 1204-1211 (2003).
3. Callahan RA, Coffey DC, Chen D, Clark NA, Rumbles G, Walba DM. Charge Generation Measured for Fullerene-Helical Nanofilament Liquid Crystal Heterojunctions. *ACS Appl. Mater. Interfaces* **6**, 4823-4830 (2014).
4. Zep A, Salamonczyk M, Vaupotic N, Pociecha D, Gorecka E. Physical gels made of liquid crystalline B-4 phase. *Chem. Commun.* **49**, 3119-3121 (2013).
5. Chen D*, et al.* Nanoconfinement of guest materials by helical nanofilament networks of bent-core mesogens. *Soft Matter* **9**, 462-471 (2013).
6. Hough LE*, et al.* Helical nanofilament phases. *Science* **325**, 456-460 (2009).
7. Chen D*, et al.* Chirality-preserving growth of helical filaments in the B4 phase of bent-core liquid crystals. *J. Am. Chem. Soc.* **133**, 12656-12663 (2011).
8. Kim H*, et al.* Linkage-length dependent structuring behaviour of bent-core molecules in helical nanostructures. *Soft Matter* **12**, 3326-3330 (2016).
9. Walba DM, Eshdat L, Korblova E, Shoemaker RK. On the nature of the B4 banana phase: Crystal or not a crystal? *Cryst. Growth Des.* **5**, 2091-2099 (2005).
10. Yamada K*, et al.* Structural analysis of a banana-liquid crystal in the B4 phase by solid-state NMR. *J. Phys. Chem. B* **117**, 6830-6838 (2013).
11. Nakata M*, et al.* A racemic layer structure in a chiral bent-core ferroelectric liquid crystal. *Liq. Cryst.* **28**, 1301-1308 (2001).
12. Lin SC, Ho RM, Chang CY, Hsu CS. Hierarchical superstructures with control of helicity from the self-assembly of chiral bent-core molecules. *Chem.-Eur. J.* **18**, 9091-9098 (2012).
13. Niwano H, Nakata M, Thisayukta J, Link DR, Takezoe H, Watanabe J. Chiral memory on transition between the B2 and B4 phases in an achiral banana-shaped molecular system. *J. Phys. Chem. B* **108**, 14889-14896 (2004).
14. Chen D*, et al.* Diastereomeric liquid crystal domains at the mesoscale. *Nat. Commun.* **6**, 7763 (2015).
15. Tuchband MR*, et al.* Manipulating the twist sense of helical nanofilaments of bent-core liquid crystals using rod-shaped, chiral mesogenic dopants. *Liq. Cryst.* **43**, 1083-1091 (2016).
16. Zhang C, Diorio N, Lavrentovich OD, Jákli A. Helical nanofilaments of bent-core liquid crystals with a second twist. *Nat. Commun.* **5**, 3302 (2014).

17. Yoon DK*, et al.* Orientation of a helical nanofilament (B4) liquid-crystal phase: Topographic Control of confinement, shear flow, and temperature gradients. *Adv. Mater.* **23**, 1962-1967 (2011).

18. Lee S*, et al.* Multidimensional helical nanostructures in multiscale nanochannels. *Langmuir* **31**, 8156-8161 (2015).

19. Kim H*, et al.* Multistep hierarchical self-assembly of chiral nanopore arrays. *Proc. Natl. Acad. Sci. U.S.A.* **111**, 14342-14347 (2014).

20. Lee S*, et al.* Physico-chemical confinement of helical nanofilaments. *Soft Matter* **11**, 3653-3659 (2015).

21. Ryu SH*, et al.* Nucleation and growth of a helical nanofilament (B4) liquid-crystal phase confined in nanobowls. *Soft Matter* **11**, 7778-7782 (2015).

22. Tsai E*, et al.* A modulated helical nanofilament phase. *Angew. Chem. Int. Ed.* **52**, 5254-5257 (2013).
